# Systemic T Cell Receptor Profiling Reveals Adaptive Immune Activation and Potential Immune Signatures of Diagnosis and Brain Atrophy in Epilepsy

**DOI:** 10.1002/acn3.70203

**Published:** 2025-10-07

**Authors:** Yong‐Won Shin, Sang Bin Hong, Yong Woo Shin, Inpyeong Hwang, Jaeseong Oh, Jihyeon Choi, Narae Kim, Jangsup Moon, Keun‐Hwa Jung, Kyung‐Il Park, Ki‐Young Jung, Kon Chu, Sang Kun Lee

**Affiliations:** ^1^ Department of Critical Care Medicine Seoul National University Hospital Seoul Republic of Korea; ^2^ Laboratory for Neurotherapeutics, Center for Medical Innovation, Biomedical Research Institute Seoul National University Hospital Seoul Republic of Korea; ^3^ Center for Hospital Medicine Seoul National University Hospital Seoul Republic of Korea; ^4^ Department of Neurology Seoul National University Hospital, Seoul National University College of Medicine Seoul Republic of Korea; ^5^ Department of Neurology Inha University Hospital Incheon Republic of Korea; ^6^ Department of Radiology Seoul National University Hospital, Seoul National University College of Medicine Seoul Republic of Korea; ^7^ Department of Pharmacology Jeju National University College of Medicine Jeju Republic of Korea; ^8^ Jeju National University Hospital Clinical Research Institute Jeju Republic of Korea; ^9^ Department of Genomic Medicine Seoul National University Hospital Seoul Republic of Korea; ^10^ Department of Neurology Seoul National University Hospital Healthcare System Gangnam Center Seoul Republic of Korea

**Keywords:** biomarker, brain atrophy, epilepsy, machine learning, TCR repertoire

## Abstract

**Objective:**

Epilepsy is increasingly associated with immune dysregulation and inflammation. The T cell receptor (TCR), a key mediator of adaptive immunity, shows repertoire alterations in various immune‐mediated diseases. The unique TCR sequence serves as a molecular barcode for T cells, and clonal expansion accompanied by reduced overall TCR repertoire diversity reflects adaptive immune activation. We investigated peripheral TCR repertoire changes in epilepsy and their association with disease severity and brain atrophy.

**Methods:**

We profiled TCR α/β chain repertoires from peripheral blood mononuclear cells of 100 individuals, including 45 patients with epilepsy (14 with well‐controlled epilepsy, 22 with drug‐resistant epilepsy [DRE], and 9 with neuroinflammation‐associated epilepsy [NIE]) and 55 unmatched healthy controls. NIE included new‐onset epilepsy following possible autoimmune or infectious neuroinflammation. We comprehensively evaluated clonotype distribution, diversity, interindividual sharing, and V/J gene usage. Machine learning models evaluated the diagnostic potential of TCR repertoire features. Brain volumes were measured by MRI and correlated with TCR repertoire characteristics.

**Results:**

Patients with epilepsy showed significantly reduced TCR diversity, particularly in DRE or NIE. They also showed distinct patterns of V and J gene usage and decreased interindividual sharing of epilepsy‐associated clonotypes. Machine learning models incorporating V/J usage and public clonotypes distinguished patients with epilepsy from controls with a mean classification accuracy of 0.80 (95% bias‐corrected and accelerated bootstrap confidence interval (BCa CI), 0.69–0.86) and the area under the curve of 0.80 (95% BCa CI, 0.70–0.87). TCR diversity correlated with seizure frequency among patients without daily seizures or clinical evidence of neuroinflammation. Brain atrophy, notably in the thalamus and basal ganglia, was also associated with TCR repertoire alterations and specific V/J gene usage patterns.

**Interpretation:**

Peripheral TCR repertoire profiling reveals that systemic immune dysregulation is present in epilepsy and is associated with neurodegeneration. Our findings highlight the peripheral TCR repertoire as a disease‐relevant immune signature with the potential to non‐invasively interrogate epilepsy status and guide therapeutic interventions.

## 1 Introduction

Epilepsy is a complex neurological disorder originating from diverse etiologies, including genetic predisposition and acquired neurological insults. Beyond its neuronal basis, increasing evidence highlights its critical interplay with the immune system [1]. Several studies have demonstrated the involvement of neuroinflammatory mechanisms in epileptogenesis and seizure generation, as well as their potential as biomarkers and therapeutic targets [2, 3, 4].

Studies reveal diverse immune cell infiltration in epileptic brain tissue [5, 6, 7, 8, 9]. Xu et al. [5] found a link between antigen‐presenting and T cell infiltration and seizure frequency, while Kumar et al. [6] reported direct physical interactions between T cells and microglia, alongside pro‐inflammatory immune cell infiltration and activation of microglia. Analysis of peripheral blood mononuclear cells (PBMCs) and cerebrospinal fluid (CSF) from epilepsy patients shows altered immune cell profiles, particularly T cells [10, 11]. Mass cytometry analysis of PBMCs from pediatric refractory epilepsy and autoimmune encephalitis patients demonstrated an expansion of pro‐inflammatory CD4^+^ and CD8^+^ T cell subsets, reduced inhibitory CD8^+^ T cell subsets, and disrupted regulatory interactions [12]. These findings collectively indicate a critical role for T cells in epilepsy pathophysiology.

T cells, central to adaptive immunity, recognize peptide‐major histocompatibility complex (MHC) molecules through the T cell receptor (TCR), a heterodimer of α and β chains (or γ and δ chains in a minor subset of T cells) generated by the V(D)J recombination process within the thymus [13, 14, 15]. The recombination process is a series of stochastic molecular events in which the random selection of germline V, D, and J gene segments, along with nucleotide insertions or deletions, results in a highly diverse repertoire of TCR clones capable of recognizing a broad range of peptide–MHC complexes. The complementarity‐determining region 3 (CDR3) region is the most variable part of the TCR, encompassing the VD and DJ junctions (or the VJ junction in the α chain), and plays a key role in peptide recognition [16]. Its unique sequence, along with the selected V and J gene segments, serves as a molecular barcode for T cells and is a key determinant in characterizing the TCR repertoire [17]. Clonal expansion of T cells with specific TCRs reduces the diversity of the TCR repertoire within a finite sample size, serving as a marker of an enhanced adaptive immune response, as seen in infections, cancer, and autoimmune diseases [17, 18].

Emerging evidence suggests that alterations in the TCR repertoire may also occur in epilepsy; however, the extent and implications of these changes remain poorly understood. Recent technological advancements, such as high‐throughput sequencing, sophisticated TCR library generation protocols, and computational models, enable detailed investigations into TCR repertoire dynamics [17, 19]. In this study, we comprehensively analyzed multiple aspects of TCR repertoire alterations in epilepsy and explored their associations with the treatment response, seizure burden, and brain atrophy. We further integrated TCR profiles with neuroimaging and machine learning to evaluate their potential as non‐invasive biomarkers for diagnosis, disease severity, and neurodegeneration.

## 2 Methods

### 2.1 Study Population

We enrolled patients admitted to the Department of Neurology at Seoul National University Hospital for epilepsy evaluation between December 2020 and March 2023. Eligible participants had a relevant clinical history and a diagnosis of epilepsy confirmed by EEG. Epilepsy was categorized into three mutually exclusive groups: (1) well‐controlled epilepsy (WCE), defined as seizures managed with two or fewer antiseizure medications; (2) drug‐resistant epilepsy (DRE), defined as requiring more than two antiseizure medications; and (3) neuroinflammation‐associated epilepsy (NIE), defined as epilepsy with clinical or historical features suggestive of a preceding neuroinflammatory process, in the absence of detectable neuronal autoantibodies. This included: (i) new‐onset epilepsy following possible autoimmune encephalitis meeting the criteria proposed by Graus et al. [20], (ii) a history of new‐onset status epilepticus, regardless of refractoriness [21]; or (iii) epilepsy onset within several years following a documented neuroinflammatory event, such as meningitis or meningoencephalitis. To minimize potential TCR repertoire biases from active systemic inflammation, we included only ambulatory individuals in the epilepsy group. Patients diagnosed with antibody‐associated autoimmune encephalitis, including anti‐N‐methyl‐d‐aspartate receptor (NMDAR) and anti‐leucine‐rich glioma‐inactivated 1 (LGI1) encephalitis, were excluded, as these conditions involve an established adaptive immune response with autoantibody production and are therefore expected to significantly affect TCR repertoire. A healthy control population without recent infectious history or active disease was separately enrolled. Details of the study inclusion process are summarized in Figure S1. The study was approved by the Institutional Review Board of Seoul National University Hospital, and written informed consent was obtained from all participants or their legal representatives.

### 2.2 Sample Processing and TCR Repertoire Data Generation

PBMCs were isolated from whole blood using SepMate with Ficoll‐Paque, following the manufacturer's instructions (STEMCELL Technologies, Vancouver, Canada). Bulk RNA extracted from PBMCs was used for TCR repertoire analysis. To enhance the sensitivity of TCR sequence detection and minimize polymerase chain reaction (PCR) and sequencing errors, we generated TCR libraries using a previously established protocol based on the rapid amplification of 5′ complementary DNA ends (5′ RACE) method, incorporating unique molecular identifiers (UMIs) [22, 23]. Briefly, UMIs were introduced during complementary DNA synthesis of TCRs via the 5′ RACE method. A two‐step PCR was then performed using TCR chain‐specific primers, with sample‐specific dual indexes incorporated during the second PCR step. To achieve sufficient repertoire depth and minimize amplification bias, each PCR step was conducted in triplicate using 700–1500 ng of total RNA, and the resulting products were pooled before sequencing. Illumina adaptors were ligated using the TruSeq DNA Nano Library Prep Kit, and libraries were sequenced on a NovaSeq 6000. Raw FASTQ data were processed using the MiGEC pipeline [24] (version 1.2.9) with collision filter and force‐overseq parameters set to 5 to obtain UMI‐consensus sequences after error correction. MiXCR [25] (version 4.0.0) was then used with default parameters for mapping to the reference, assembling clonotypes, and obtaining the TCR repertoire data. To further ensure data quality and reliability, we included only sequences that satisfied the following criteria: (1) containing TCRα (TRA) and β (TRB) chain V/J genes and a complete in‐frame CDR3 sequence; (2) having V/J genes that were not pseudogenes or open reading frame genes; (3) having a CDR3 sequence length of 7 to 23 amino acids; (4) starting with a cysteine residue and ending with phenylalanine or tryptophan, with no stop codons. To ensure comparability across samples, TCR repertoire data were downsampled separately for each chain (TRA and TRB) to 50,000 clones based on UMI counts. Subjects with fewer than 50,000 clones in their final TRA or TRB repertoires were excluded from the analysis (Figure S1). The Immunarch package [26] (version 0.9.1) was used to manage and analyze the TCR repertoire data.

### 2.3 TCR Repertoire Metrics

To assess the diversity of TCR repertoire, we calculated several metrics that reflect how broadly and evenly clonotypes are distributed within each sample. Higher diversity indicates a more balanced and heterogenous immune repertoire, whereas lower diversity and increased clonality suggest antigen‐driven clonal expansion. We quantified repertoire diversity using the following metrics: richness *S*(*X*), defined as the number of unique clonotypes; Shannon entropy *H*(*X*), which accounts for both the richness and evenness of the distribution; and clonality *C*(*X*), calculated as the inverse normalized entropy to quantify the extent of clonal expansion. These were calculated using the following established formulas:

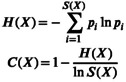

where *p*_*i*_ denotes the proportion of each clonotype *i*. Additionally, the diversity 50 (D50) index was computed, representing the minimum number of unique TCR clonotypes accounting for 50% of the total repertoire. A lower D50 value indicate that a small number of clonotype dominate the repertoire, reflecting reduced diversity and potential clonal expansion. The proportion of highly stimulated clones was estimated using the powerTCR package [27], which analyzes clone size distribution by fitting it to a discrete gamma‐generalized Pareto distribution (GPD) spliced threshold model, with default parameter settings. Clones above this data‐driven threshold are considered highly stimulated and may reflect antigen‐driven clonal proliferation. Estimating their proportion provides a summary of immune skewness and clonal dominance.

### 2.4 TCR Repertoire Similarity Evaluation

We next evaluated the similarity of TCR repertoires between individuals. The degree of repertoire overlap can reflect convergent immune responses or the presence of disease‐associated shared clonotypes. To evaluate the similarity of TCR repertoires, three indices were employed: the number of shared clonotypes, the Jaccard index, and the Morisita overlap index. The Jaccard index, measuring the ratio of shared unique clonotypes between two repertoires, was calculated as:

$$J\left(A,B\right)=\frac{\left|A\cap B\right|}{\left|A\cup B\right|}$$

where *A* and *B* are the sets of unique clonotypes from two repertoires. The Morisita overlap index, which accounts for both the presence and frequency of clonotypes, was computed as:

$$C_{M}\left(A,B\right)=\frac{2\sum_{i}\left(n_{\mathrm{iA}}\cdot n_{\mathrm{iB}}\right)}{\left(\frac{\sum_{i}n_{\mathrm{iA}}^{2}}{N_{A}^{2}}+\frac{\sum_{i}n_{\mathrm{iB}}^{2}}{N_{B}^{2}}\right)\cdot N_{A}\cdot N_{B}}$$

where *n*_*iA*_ and *n*_*iB*_ are the counts of i‐th clonotype, and *N*_*A*_ and *N*_*B*_ are the total counts of clonotypes in repertoires *A* and *B*, respectively. This index measures similarity by weighting the abundance of shared clonotypes while reducing sensitivity to rare clonotypes. All three overlap metrics were calculated using the “repOverlap” function from the immunarch package [26]. Hierarchical clustering of the number of shared clonotypes was visualized using the pheatmap package, with both rows and columns hierarchically clustered using the “hclust” function with the “complete” linkage method.

### 2.5 TCR Sequence Generation Probability

The generation probability (*P*_gen_) of each TCR clonotype, which represents the likelihood that it is to be generated through the stochastic V(D)J recombination process, is inherently non‐uniform and can be estimated using computational algorithms. This influences the likelihood of detecting a given clonotype in the naïve TCR repertoire. However, antigen‐driven selection and clonal expansion during adaptive immune responses can alter this distribution and create a gap between the theoretical TCR distribution based on *P*_gen_ and the actual observed repertoire. We estimated the *P*_gen_ of each TCR clonotype using the Optimized Likelihood estimate of immunoGlobulin Amino‐acid sequences (OLGA) algorithm [28] with default parameters. The calculation was performed by incorporating both the CDR3 sequence and V/J gene segment information.

### 2.6 Machine Learning for Prediction of Epilepsy Based on TCR Repertoire

We trained nine machine learning models, including random forest (with 500 trees), naïve Bayes, XGBoost (trained for 100 rounds with a logistic objective function), *k*‐nearest neighbors (*k* = 5), support vector machine with linear and radial basis function kernels, elastic net (with an equal mixture of Lasso and Ridge regularization and cross‐validation to select the optimal regularization parameter *λ*), linear discriminant analysis, and logistic regression, to distinguish epilepsy from control based on TCR repertoire features. A total of 18 dataset combinations (3 × 3 × 2) were generated by considering (1) whether the TRA repertoire, TRB repertoire, or both were used, (2) whether V‐J gene usage, clonotype frequency, or both were included, and (3) whether age and sex information were included. Features from each dataset were filtered via Wilcoxon test (*p* < 0.01) and subsequently used to train nine algorithms, resulting in 162 models. Prior to model training, feature scaling was performed via standardization. Model performance was assessed using leave‐one‐out cross‐validation with 1000 bootstrap iterations. Performance metrics including accuracy, precision, recall, sensitivity, and F1‐score were calculated based on predicted class labels, while the area under the curve (AUC) was derived from predicted probability values. For models requiring a classification threshold (naïve Bayes, XGBoost, elastic net, and logistic regression), test samples were classified as epilepsy if the predicted probability was ≥ 0.5. Confidence intervals (CIs) for bootstrapped performance metrics were estimated using the bias‐corrected and accelerated (BCa) bootstrap method to ensure accurate interval estimation. Model training and evaluation were implemented in R using the packages caret, randomForest, e1071, xgboost, class, glmnet, MASS, boot, and pROC, with default settings unless otherwise specified. To evaluate the importance of individual features and their impact on model predictions, Shapley Additive Explanations (SHAP) were employed using the DALEX package. A model explainer was created using the “explain” function. SHAP values were then calculated with the “predict_parts” function, which internally uses the “shap” function from the iBreakDown package.

### 2.7 MRI Volumetric Analysis

From the enrolled subjects, we included epilepsy patients with MRI scans free of major structural lesions in the volumetric analysis (*n* = 21). Three patients with cerebromalacia and one with a history of surgical removal of a dysembryoplastic neuroepithelial tumor (DNET) were excluded. High resolution (1 mm‐thick) 3D T1‐weighted MRI scans were acquired using a 3.0 T scanner. Automated segmentation and volumetric analysis were performed using NeuroQuant (version 3.1; CorTechs Labs, San Diego, CA, USA). To adjust for individual variations in brain size, volumes were normalized as a percentage of intracranial volume (ICV), enabling standardized comparisons across subjects. Volumetric analyses were performed at three levels: (1) whole brain volume, (2) major lobes (frontal, parietal, temporal, occipital), the cingulate cortex, and key subcortical structures (thalamus and basal ganglia), and (3) cortical subregions as segmented by NeuroQuant.

### 2.8 Statistical Analysis

Non‐parametric tests, including the Fisher exact test, Wilcoxon rank‐sum test, and Kruskal‐Wallis test with post hoc tests using Bonferroni correction for multiple comparisons, were used as appropriate. Spearman's rank correlation analysis was also used to assess the association between variables. The Jonckheere‐Terpstra Test was applied to evaluate statistically significant trends in continuous variables. Corrected *p* values for multiple comparisons other than post hoc tests were calculated using the Benjamini‐Hochberg method. All statistical analyses were performed using R. For dimensionality reduction, principal component analysis (PCA) was conducted using the “prcomp” function from the base stats package and the “get_pca_var” function from the factoextra package. To control for potential confounding effects, a linear regression model was used to adjust for age and sex, and the adjusted data were then employed in subsequent analyses. Alternatively, partial correlation analysis was performed using the “pcor.test” function from the ppcor package. To correct for multiple variables in partial correlation analysis, LASSO (Least Absolute Shrinkage and Selection Operator) regression was applied to select the most relevant predictors. To handle age‐related variables (age, age at seizure onset, and duration of epilepsy), PCA was applied to derive uncorrelated components prior to LASSO‐based variable selection. LASSO regression was performed using the “cv.glmnet” function in the glmnet package with 10‐fold cross‐validation, selecting the optimal parameter *λ* based on the minimum cross‐validation error (“lambda.min”) to retain variables with non‐zero coefficients.

## 3 Results

### 3.1 Clonal Distribution and Diversity of TCR Repertoire

A total of 100 subjects were included in this study, comprising 45 patients with epilepsy and 55 controls (Figure S1). The epilepsy group was younger than the control group, although there was no significant difference in sex distribution. The clinical characteristics are summarized in Table S1.

Given that each TCR sequence acts as a molecular barcode that reflects T cell identity and antigen exposure, we first investigated how clonotype composition and repertoire diversity differ between epilepsy and control groups. Clonotype distribution showed a significantly decreased number of unique clonotypes in the epilepsy group compared to controls, consistent with clonal expansion, as the total number of T cell clones was equal across all samples (Figure S2A). This decrease was more pronounced in clonotypes with longer CDR3 lengths. While the mean CDR3 length of each individual TCR repertoire was shorter in the epilepsy group (Figure S3A), the group‐level difference in median CDR3 length across individual repertoires did not reach statistical significance, although it showed a similar trend (*p* = 0.06). The number of clones for each CDR3 length (i.e., the sum of clone counts for all clonotypes of that length) was comparable between groups (Figure S2B), indicating that clonotypes with longer CDR3s in the epilepsy group, though fewer in number, were represented by more cells (i.e., had higher clone counts), suggesting greater clonal expansion. This finding is particularly notable given that TCRs with longer CDR3 lengths typically exhibit both a lower probability of generation and are more private (i.e., less shared across individuals), as reflected by their lower frequencies in the CDR3 length distribution and supported by prior studies [29, 30]. Their expansion in the epilepsy group therefore suggests a more selective and potentially antigen‐driven clonal response. Interestingly, when restricting the analysis to highly expanded clonotypes (defined as those represented by more than four clones per subject), the number of clonotypes with shorter CDR3 lengths was markedly higher in the epilepsy group compared to controls (Figure S2C,D). This enrichment suggests a more dominant expansion of shorter‐CDR3 clonotypes within the highly expanded repertoire in epilepsy, consistent with previous studies demonstrating a more favorable peptide–MHC binding affinity for TCRs with shorter CDR3 lengths [30].

**FIGURE 1:**
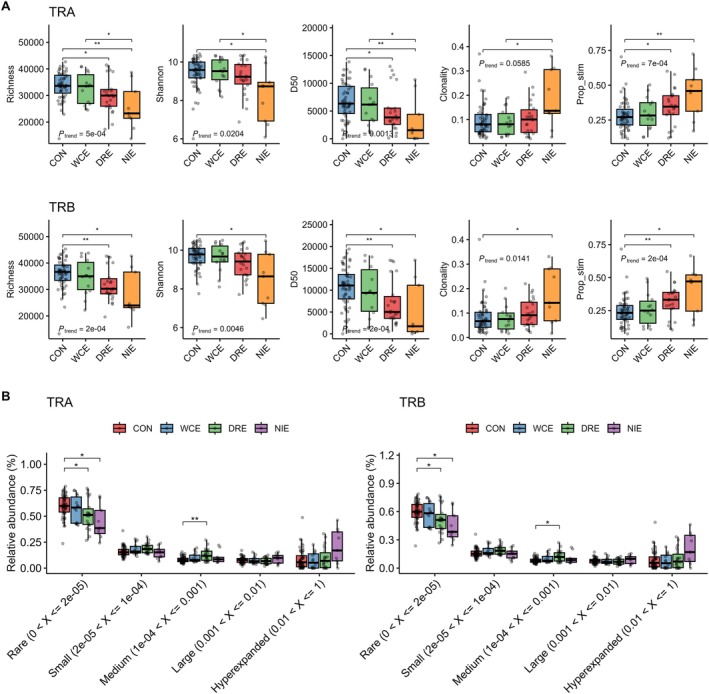
TCR repertoire diversity and clonal frequency distribution across epilepsy subgroups. (A) Comparison of five diversity metrics across control, WCE, DRE, and NIE. Pairwise comparisons are performed using the Wilcoxon rank‐sum test (unadjusted), and overall trends are assessed with the Jonckheere‐Terpstra test (*p* values annotated). (B) Comparisons of the relative abundance across five clonal frequency categories for TRA and TRB. Adjusted *p* values for each comparison are displayed at the top of each clonal frequency category barplot. Statistical significance is indicated as follows: *p < 0.05, **p < 0.01. CDR3 = complementarity‐determining region 3; CON = control; D50 = the minimum number of clones accounting for 50% of the repertoire; DRE = drug‐resistant epilepsy; NIE = neuroinflammation‐associated epilepsy; Prop_stim = proportion of highly stimulated clones; Shannon = Shannon diversity index; TRA = T cell receptor α chain; TRB = T cell receptor β chain; WCE = well‐controlled epilepsy.

As clonal expansion of specific T cells leads to a reduction in diversity within a bounded TCR repertoire, we next evaluated repertoire diversity to compare repertoire‐level changes between the control group and the epilepsy group, including its clinical subgroups. TCR repertoire diversity, assessed using several indices, was generally lower in the epilepsy group, with a corresponding increase in the proportion of expanded T cell clones (Figure 1A and Figure S4). This difference was notable given the known age‐related decline in TCR diversity [31, 32], as the epilepsy group was younger than the control groups (Table S1) and still exhibited reduced diversity after age adjustment (Figure S4). The difference was primarily driven by a decreased proportion of rare clonotypes and an increased proportion of clonotypes of medium abundance (Figure 1B). While the WCE group did not differ significantly in TCR diversity compared to controls, the DRE and NIE groups exhibited a progressive trend toward clonal expansion and reduced diversity (Figure 1A).

### 3.2 V and J Gene Usage in Epilepsy

**FIGURE 2:**
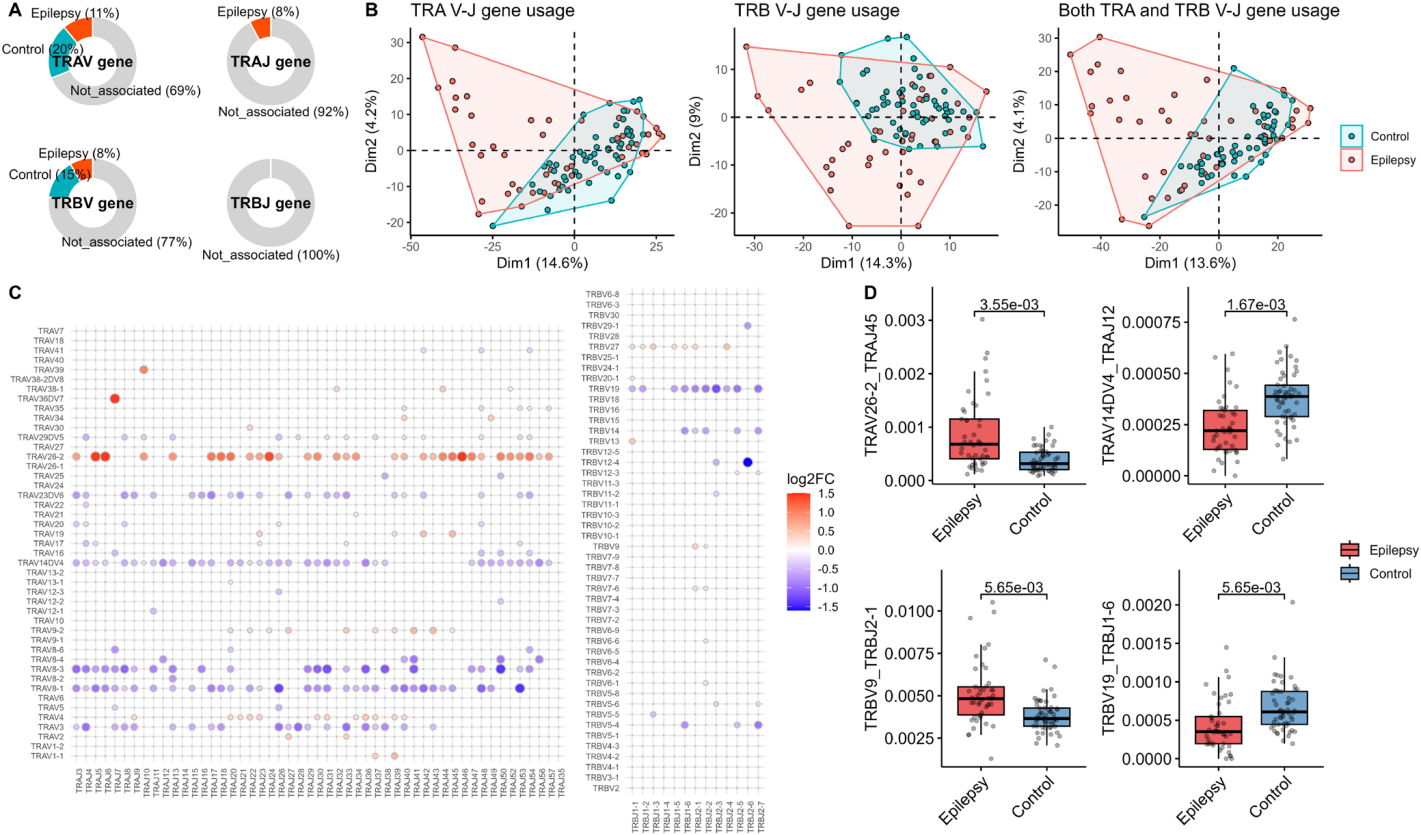
Group differences in V/J gene usage of the TCR repertoire. (A) Proportion of V and J gene segments with significantly different usage frequencies between epilepsy and control groups, categorized based on whether the median frequency is higher in epilepsy. (B) Principal component analysis plot of the first and second principal components based on combined V‐J gene usage frequencies, with points grouped by epilepsy and control. Axis labels indicate the principal components and the percentage of variance explained by each. (C) Log₂ fold‐change differences in significantly altered V‐J gene usage, with red indicating higher usage in epilepsy and blue indicating higher usage in controls. (D) Boxplots of selected V‐J gene usages showing the most significant differences in each TRA and TRB repertoire, with two V‐J pairs exhibiting higher usage in epilepsy and two showing higher usage in controls. All statistical significance is based on FDR‐corrected *p* values. FDR = false discovery rate; TCR = T cell receptor; TRA = T cell receptor α chain; TRB = T cell receptor β chain.

We analyzed differences in V and J gene usage, as well as combined V‐J gene usage, between the epilepsy and control groups. Several V gene segments of TRA (TRAV) and TRB (TRBV), as well as J gene segments of TRA (TRAJ), showed significant differences in frequency between the two groups (Figure 2A and Table S2). There were also quite a number of V and J gene pairs with significant differences in their frequency between the groups (Figure 2C,D). PCA based on combined V‐J gene usage frequencies per subject distinguished epilepsy from control groups, though with some individual overlap (Figure 2B). Additional PCA separating WCE, DRE, and NIE subgroups did not reveal distinct clustering among the epilepsy subgroups, indicating that the separation is not driven by any specific epilepsy subgroup (Figure S5).

### 3.3 Clonotype Sharing Among Subjects

**FIGURE 3:**
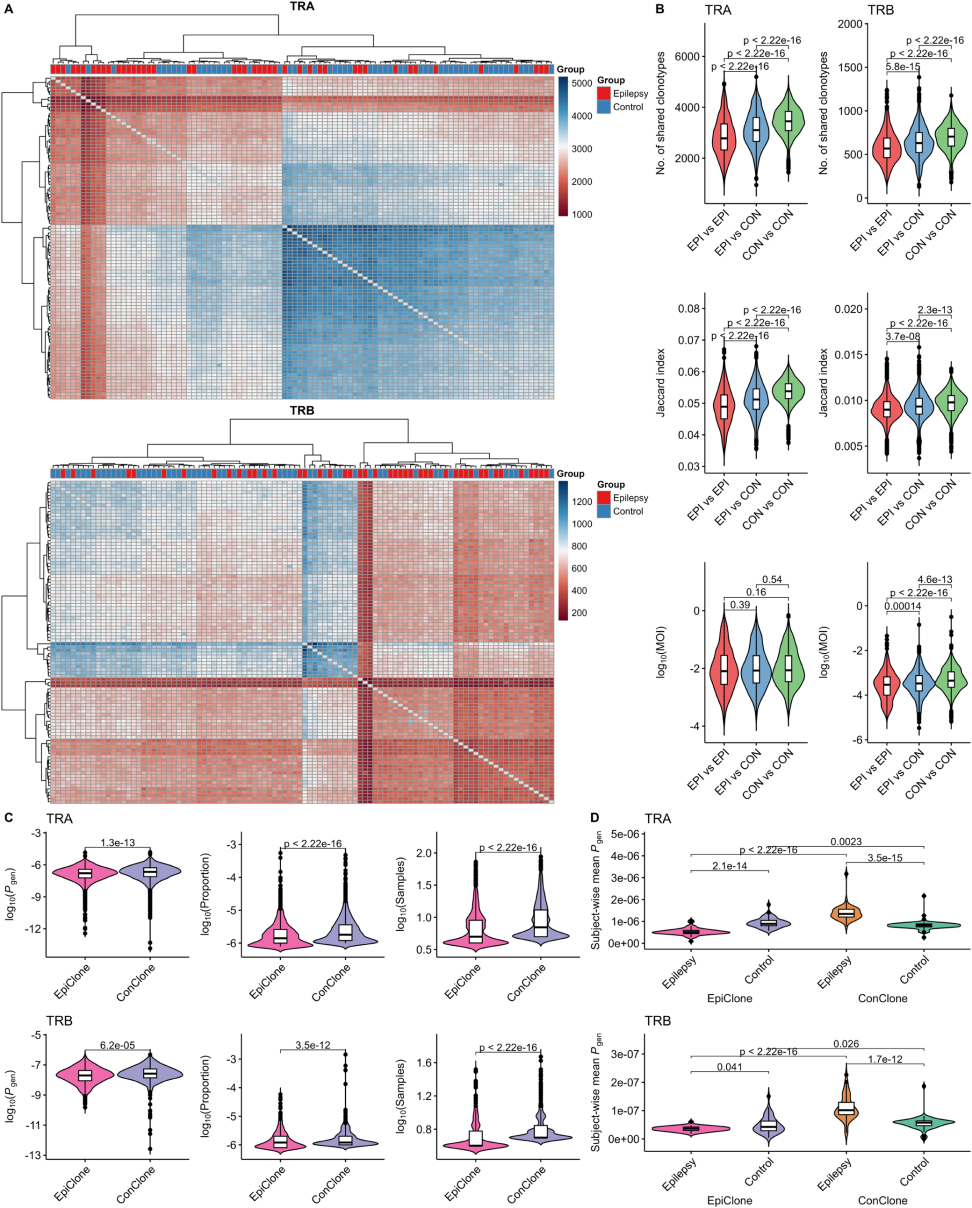
Clonotype sharing and overlap between epilepsy and control groups. (A) Heatmap displaying the number of shared clonotypes between samples, with hierarchical clustering applied to both rows and columns. (B) Comparison of clonotype overlap between samples using multiple overlap indices, categorized into epilepsy versus epilepsy, epilepsy versus control, and control versus control groups. (C) Comparison of *P*_gen_, proportion in the repertoire, and the number of samples sharing each clonotype between EpiClones and ConClones. (D) Comparison of mean *P*_gen_s of EpiClones and ConClones across and within epilepsy and control groups. CON = control; ConClone = clonotypes with significantly higher frequency in the control TCR repertoire compared to epilepsy; EPI = epilepsy; EpiClone = clonotypes with significantly higher frequency in the epilepsy TCR repertoire compared to controls; MOI = Morisita overlap index; *P*_gen_ = generation probability of each TCR clonotype.

We next analyzed the overlap of both the TRA and TRB repertoires between subjects. The number of shared clonotypes revealed a tendency for clustering within the epilepsy and control groups (Figure 3A and Figure S6). In addition to the number of shared clonotypes, both the Jaccard index and the Morisita overlap index showed a trend toward reduced clonotype sharing in comparisons involving epilepsy subjects (Figure 3A,B). To further investigate this pattern, we compared *P*_gen_ values of clonotypes predominantly found in epilepsy (termed EpiClones, defined as those with significantly higher frequencies in the epilepsy group than in the control group by Wilcoxon rank‐sum test and a greater total number of clones in the epilepsy repertoire) with those of clonotypes predominant in controls (termed ConClones, defined similarly but with higher clone counts in the control repertoire). EpiClones exhibited significantly lower *P*_gen_ values compared to ConClones. This suggests that the reduced clonotype overlap observed among epilepsy subjects and between epilepsy and control subjects is, at least in part, attributable to the lower generation probability of EpiClones (Figure 3C). Furthermore, the proportion of EpiClones within the total epilepsy repertoire was lower than that of ConClones in the control repertoire (Figure 3C). The number of samples sharing EpiClones was also significantly lower than that for ConClones, indicating that EpiClones are less public (Figure 3C). At the individual level, the mean *P*_gen_ of clones classified as EpiClones was lower in epilepsy subjects, while the mean *P*_gen_ of ConClones was lower in control subjects (Figure 3D). Additionally, EpiClones detected in control subjects tended to have a higher *P*_gen_ than those found in epilepsy subjects, while ConClones found in epilepsy subjects exhibited higher *P*_gen_ values than those classified as ConClones in control subjects (Figure 3D).

### 3.4 Predictability of Epilepsy Using TCR Repertoire With Machine Learning

The characterization of the TCR repertoire in epilepsy strongly suggests that it can serve as a surrogate marker for predicting epilepsy. To evaluate this, we employed nine machine learning models for classification. Performance differed by model and input features, with the best results achieved using V‐J usage from both TRA and TRB repertoires.

**FIGURE 4:**
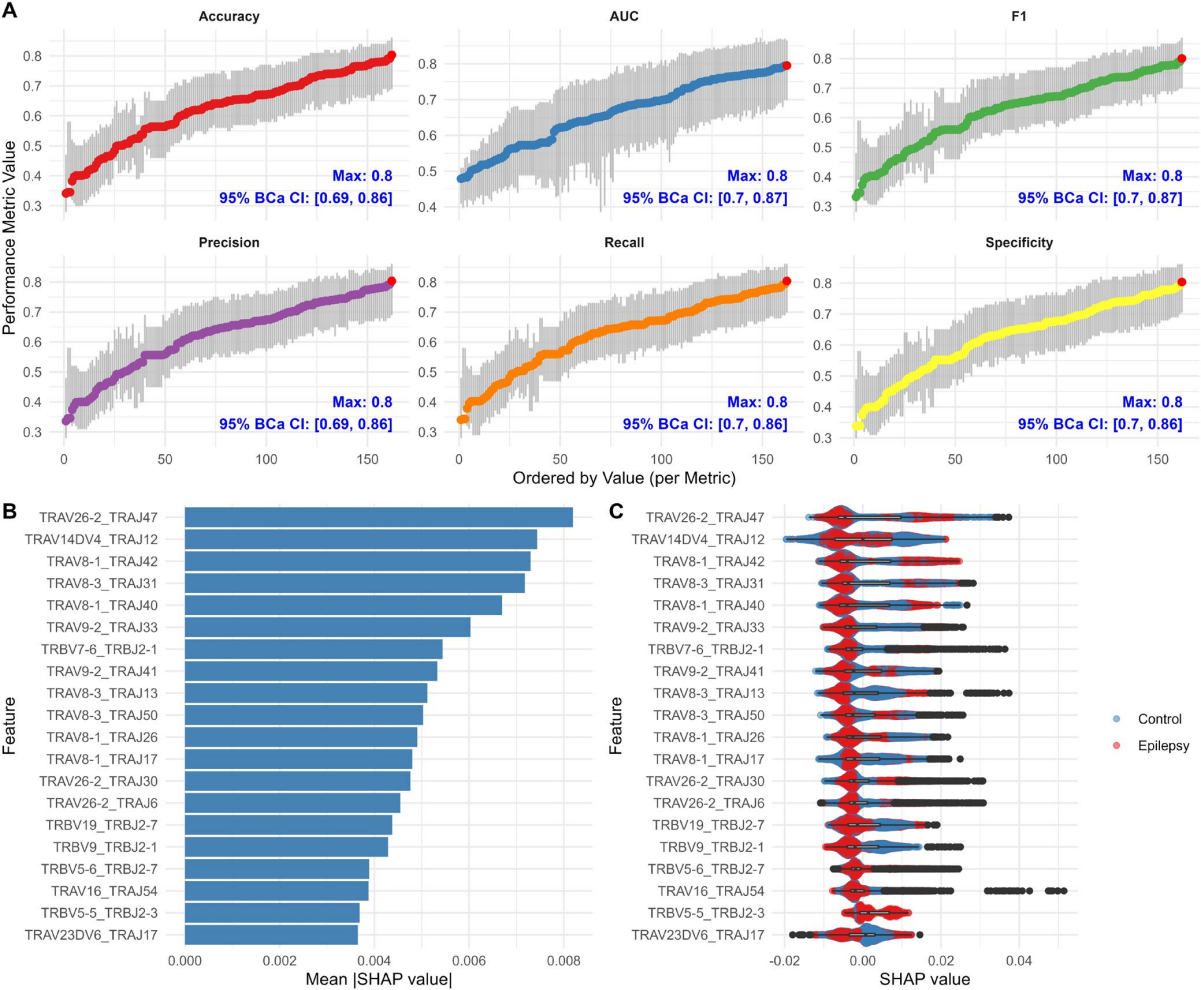
Machine learning classification performance for epilepsy. (A) Performance metrics of leave‐one‐out cross‐validation. Each point represents a metric value, with gray lines indicating the 95% BCa CI. Red points mark the highest metric values. The 95% BCa CI and corresponding maximum values are displayed in the bottom right of each plot. (B) Top 20 V‐J gene features ranked by mean absolute SHAP values, indicating their importance in classification. (C) Distribution of SHAP values for V‐J genes, showing their contribution to model predictions and variability. AUC = area under the curve; BCa CI = bias‐corrected and accelerated confidence interval; F1 = F1‐score; SHAP = Shapley Additive Explanations.

Model performance varied depending on the model and dataset used. Nonetheless, consistently high performance across multiple metrics was achieved by selecting appropriate combinations (Figure 4A). The random forest model using combined TRA and TRB V‐J gene usage data yielded the best performance for all metrics except AUC, for which the highest value was obtained using TRA V‐J gene usage alone. Notably, age, sex, and clonotype frequency information were not included in either of these best‐performing models. To further interpret the model predictions, we conducted SHAP analysis on the best‐performing model for most performance metrics—the random forest model with TRA and TRB combined V‐J gene usage data. Based on SHAP values, the most influential features were dominated by TRAV combinations, with only five of them being TRBV combinations among the top 20 features (Figure 4B). For the top 20 features, there was substantial variability in SHAP values, with their dependence on feature values showing monotonic, U‐shaped, or other variable non‐monotonic patterns (Figure 4C and Figure S7), which might reflect the heterogeneous nature of epilepsy and complex immune responses. The total feature impact, calculated as the sum of mean absolute SHAP values for all features, was 0.631, with the impact of the top 20 features being 0.107, accounting for approximately 17% of the total feature impact. This indicates that the model relies on a broad range of features rather than just a few dominant ones. In our evaluation of multiple models across various datasets, the TRA repertoire contributed more significantly to higher performance, and V‐J gene usage was more informative than clonotype frequency (Figure S8A,B). The inclusion of age and sex information, however, did not substantially improve performance (Figure S8C). Among the nine models evaluated on diverse datasets, the random forest model consistently achieved the highest average performance across all metrics, with relatively low variability in performance between datasets (Figure S8D).

### 3.5 Association Between Clinical Characteristics and TCR Repertoire

**FIGURE 5:**
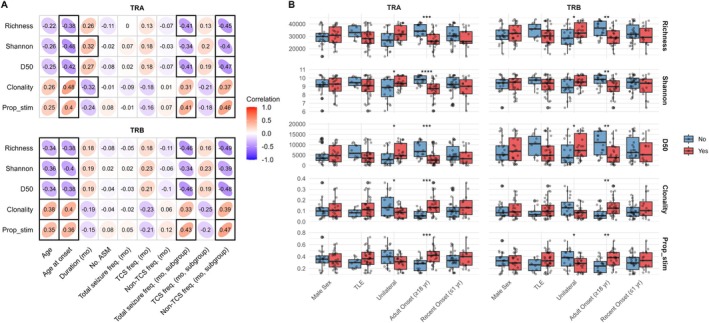
Association between TCR repertoire diversity metrics and demographic/clinical characteristics in the epilepsy group. (A) Correlation matrix showing the relationship between demographic/clinical variables (*x*‐axis) and TCR repertoire diversity metrics (*y*‐axis). Each box displays the correlation coefficient, with the shape and eccentricity of the ellipse representing the strength and direction of the correlation. Statistically significant (*p* < 0.05) correlations are highlighted with thick black squares. (B) Comparison of TCR repertoire diversity metrics across categorical clinical variables. Statistical significance is indicated as follows: *p < 0.05, **p < 0.01, ***p < 0.001. BMI = body mass index; D50 = the minimum number of clones accounting for 50% of the repertoire; freq. = frequency; No. ASM = number of antiseizure medications at the time of study inclusion; Prop_stim = proportion of highly stimulated clones; Shannon = Shannon diversity index; subgroup = subjects not categorized in neuroinflammation‐associated epilepsy and without daily seizures; TCR = T cell receptor; TCS = tonic–clonic seizure; TRA = T cell receptor α chain; TRB = T cell receptor β chain.

To further contextualize our findings, we analyzed the demographic and clinical characteristics of epilepsy subjects in relation to both TRA and TRB repertoires. Among continuous variables, age, age at seizure onset, and duration of epilepsy were significantly associated with TCR repertoire diversity metrics (Figure 5A). For categorical variables, unilateral involvement and adult onset were associated with TCR repertoire diversity (Figure 5B). Due to the limited number of subjects, we did not compare TCR repertoire diversity across etiologies; however, structural epilepsy, hippocampal sclerosis, and focal cortical dysplasia type IIa showed a potential trend toward reduced diversity compared to other etiologies, except for NIE (Figure S9).

Regarding seizure frequency, the initial analysis did not show a significant correlation. However, a subset of NIE patients exhibited reduced diversity and clonal expansion despite infrequent seizures, whereas patients with daily seizures tended to show reduced clonal expansion with increased diversity (Figure S10). We hypothesized that in this subset of NIE patients, elevated clonal proliferation may be primarily driven by the underlying encephalopathy, resulting in a less pronounced link to seizure activity compared to other epilepsy patients. In contrast, frequent seizures in patients with daily seizures may primarily result from aberrant network formation rather than ongoing neuroinflammation. More broadly, TCR repertoire clonal proliferation may persist for a period before eventually declining, given the higher diversity observed in patients with younger onset and longer epilepsy duration (Figure 5A). After excluding patients with NIE and daily seizures, TCR repertoire diversity metrics were significantly correlated with seizure frequency (Figure 5A and Figure S10). This correlation mostly remained significant after adjusting for age, age at seizure onset, duration of epilepsy, sex, and unilateral involvement, yielding generally higher correlation coefficients for total seizure frequency (Table S3). This finding aligns with a previous study showing increased T cell infiltration in patients with higher seizure frequency [5], suggesting that seizure activity may be related to T cell activity and reflected in systemic TCR repertoire bias.

**FIGURE 6:**
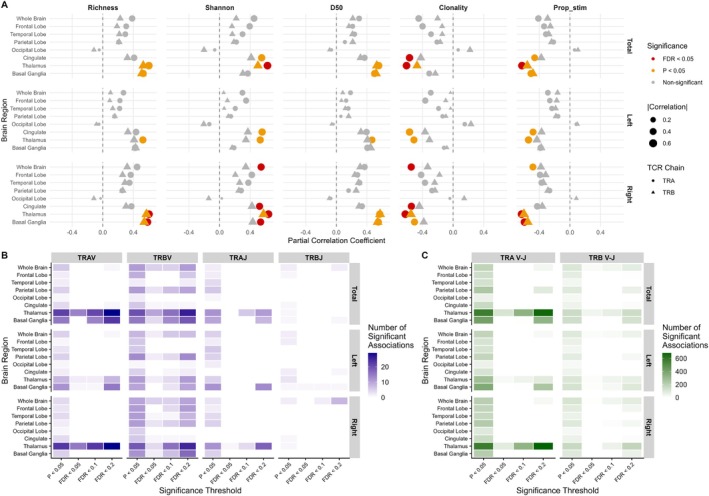
Association between the percentage of intracranial volume, TCR repertoire diversity metrics, and V/J gene usage. (A) Partial correlations between the percentage of intracranial volume and TCR repertoire diversity metrics, adjusted for age and sex. Points are colored by significance level (non‐significant, *p* < 0.05, and FDR < 0.05) and scaled by the absolute correlation coefficient. Different shapes represent TRA and TRB chains. Brain regions are analyzed separately for the total, right, and left hemispheres. (B, C) Heatmaps displaying the number of V/J gene segments (B) or V‐J gene segment combinations (C) whose frequency of usage is significantly associated with the percentage of intracranial volume. Four significance thresholds (*p* < 0.05, FDR < 0.05, FDR < 0.1, and FDR < 0.2) are used for visualization. Brain regions are analyzed separately for the total, right, and left hemispheres. D50 = the minimum number of clones accounting for 50% of the repertoire; FDR = false discovery rate; Prop_stim = proportion of highly stimulate clones; Shannon = Shannon diversity index; TCR = T cell receptor; TRA V‐J = TRAV‐TRAJ gene segment combinations; TRA = T cell receptor α chain; TRAJ = TRA J gene segments; TRAV = TRA V gene segments; TRB V‐J = TRBV‐TRBJ gene segment combinations; TRB = T cell receptor β chain; TRBJ = TRB J gene segments; TRBV = TRB V gene segments.

To extend our investigation, we examined whether decreased TCR diversity is also related to brain atrophy. Volumetric analysis revealed that whole‐brain atrophy was associated with TCR repertoire diversity, with the most pronounced effects observed in the thalamus, followed by the basal ganglia and cingulate cortex (Figure 6A). A substantial number of V and J gene usages were also associated with brain atrophy, particularly in the thalamus, providing further evidence of a close relationship between T cell activity and brain atrophy (Figure 6B). The percentage of ICV in several cortical subregions also showed significant associations with TCR repertoire diversity; however, none remained significant after multiple testing correction.

## 4 Discussion

The TCR repertoire in the epilepsy group exhibited altered clonal distribution, with reduced diversity and more expanded T cell clones in DRE and NIE, indicating chronic adaptive immune activation. Furthermore, the epilepsy group's TCR repertoire differed from controls in specific V/J gene usage and the degree of clonotype sharing of certain T cell subsets. Machine learning models showed the TCR repertoire's discriminative value for epilepsy diagnosis. In addition, seizure frequency correlated with TCR repertoire metrics in a subset of the study population. Brain atrophy was also associated with TCR repertoire metrics and specific V/J gene usage, indicating chronic inflammation linked to T cell activity.

In adaptive immune responses, T cells with TCRs reactive to specific epitopes undergo clonal expansion, skewing clonal distribution toward particular T cell subsets. Increased TCR clonality or stimulated T cell proportions suggest chronic adaptive immune activation in epilepsy. Previous evidence of T cell involvement in epileptic brain tissue strongly suggests that it contains antigens or epitopes that activate T cells [5, 6, 7, 8, 9]. Increased MHC expression in microglia further indicates inflammatory conditions in epileptic brain [6, 7, 33]. Several studies have reported associations between genetic variations in human leukocyte antigen (HLA) genes, the human equivalent of MHC, and immune‐mediated neurological conditions, including autoimmune encephalitis [34, 35, 36] and hypersensitivity reactions to antiseizure medications [37, 38]. Associations with common epilepsy syndromes, such as genetic generalized epilepsy and temporal lobe epilepsy, have also been suggested, though the supporting evidence remains limited [39, 40, 41]. Variation in MHC genes has been shown to influence TCR V gene usage [42], underscoring the importance of incorporating MHC genotyping in future studies to better characterize the adaptive immune response in epilepsy.

Growing evidence highlights the role of regulatory T cells (Tregs), a subset of T cells that maintain immune tolerance by controlling autoreactive immune cells, in various neurological disorders [43]. Tregs also show increased infiltration and activation in the epileptic brain [44]. These changes suggest a maladaptive or autoimmune response, supported by Treg alterations in autoimmune CNS disorders [45, 46, 47, 48]. The previously reported similarity in immune profile between DRE and autoimmune encephalitis [12], along with the known somatic mutational burden in epileptic tissue that increases the likelihood of neoantigen formation [49], further supports the involvement of antigen‐specific adaptive immune responses in epilepsy.

In this study, we identified a dual nature of the TCR repertoire in epilepsy. While we observed a distinct increase in T cell clonotypes with shorter CDR3 lengths among highly expanded clones, there was also a significant increase in T cells with longer CDR3 lengths or low *P*_gen_. This finding suggests active T cell engagement in epilepsy patients, accompanied by the co‐recruitment of private, patient‐specific T cells. The low generative probability and reduced publicness of EpiClones further support the presence of patient‐specific adaptive immune processes. Differences in diversity metrics were primarily driven by modestly expanded clones rather than hyperexpanded clones in our dataset. These characteristics of the TCR repertoire in the epilepsy group offer a nuanced understanding of T cell behavior and repertoire dynamics, thereby strengthening the evidence for adaptive immune activation in epilepsy.

Mounting evidence supports systemic inflammation as a crucial factor contributing to neurodegeneration and brain atrophy [50, 51]. In epilepsy, large‐scale population neuroimaging studies have identified extensive structural changes across the majority of cortical regions [52]. Longitudinal studies have also demonstrated widespread, progressive cortical atrophy in individuals with epilepsy, exceeding the rate of normal aging [53, 54]. Thalamic atrophy has been observed across various forms of epilepsy and is often attributed to the critical role of the thalamocortical pathway in seizure generation and propagation [55, 56]. The basal ganglia also play a crucial role in modulating seizure activity through the basal ganglia–thalamus network [57, 58]. Progressive brain atrophy originating in the basal ganglia represents a distinct subtype of brain atrophy in epilepsy, associated with more severe epilepsy manifestations [59]. Our data corroborate these findings, particularly highlighting the distinct vulnerability of subcortical structures to neuroinflammation in association with changes in TCR repertoire diversity and V/J gene usage.

In this study, we profiled both TRA and TRB repertoires using rigorous quality control criteria to accurately identify TCR clones with minimal errors, while restricting our analysis to samples with sufficient repertoire depth to ensure reliable comparisons. To our knowledge, this is the first study to comprehensively characterize the bulk TCR repertoire in epilepsy and its clinical relevance, as opposed to analyzing a selected TCR repertoire derived from a limited number of cells (e.g., through single‐cell TCR sequencing). To increase the generalizability of our findings in the epilepsy population, we did not restrict our analysis to disease entities with well‐established roles of T cells or adaptive immunity, such as Rasmussen encephalitis or autoantibody‐mediated encephalitis.

Despite its contributions to understanding immune alterations in epilepsy, the study has several limitations that warrant further investigation. First, epilepsy is a heterogeneous disorder, but we did not further investigate etiologically distinct conditions. Increasing the sample size or focusing on specific subpopulations may address this limitation. Our machine learning models, despite using a heterogeneous dataset, showed the diagnostic potential of TCR repertoire, suggesting that larger datasets and model refinement could yield more compelling evidence of its utility. Second, the TCR repertoire may be affected by other infectious or inflammatory conditions. To minimize this effect, we included only samples from subjects in stable disease conditions and identified general trends linking TCR repertoire features to disease severity. Individual variability across clinical groups does not preclude relevance, but warrants further study. Third, our data did not directly demonstrate T cell activation to specific epilepsy‐related antigens. It remains unclear whether observed clonal expansions reflect antigen‐driven responses or bystander activation. In addition, our cross‐sectional design precludes assessment of the temporal dynamics or causal directionality of TCR repertoire alterations in relation to seizures. While our findings suggest a link between uncontrolled seizures in epilepsy and chronic peripheral T cell activation, longitudinal studies with serial TCR profiling are needed to better delineate these relationships. The prominent TCR repertoire alterations observed in the NIE subgroup, where epilepsy onset follows a defined episode of immune activation, may offer indirect support for the hypothesis that immune dysregulation precedes and contributes to both epileptogenesis and systemic immune alterations. Nonetheless, our findings suggest a key role for T cell‐mediated adaptive immunity in seizures and epileptogenesis, providing a foundation for future research.

In conclusion, our findings collectively illustrate alterations in the TCR repertoire that reflect systemic immune dysregulation in epilepsy and are associated with neurodegeneration. By leveraging multiple TCR parameters, our study highlights the potential of systemic T cell profiling not only to elucidate underlying neuropathological mechanisms but also to serve as a promising non‐invasive biomarker for disease monitoring. Future work with larger cohorts and multimodal data may further refine patient stratification and support targeted therapeutic approaches.

## Author Contributions

Y.‐W.S., S.B.H., K.C., and S.K.L.: contributed to the conception and design of the study; Y.‐W.S., S.B.H., Y.W.S., I.H., J.O., J.C., N.K., J.M., K.‐I.P., K.‐Y.J., K.C., and S.K.L: contributed to the acquisition and analysis of data; Y.‐W.S., S.B.H., Y.‐W.S., I.H., J.O., J.C., N.K., J.M., K.‐H.J., K.‐I.P., K.‐Y.J., K.C., and S.K.L: contributed to drafting the text or preparing the figures.

## Conflicts of Interest

The authors declare no conflicts of interest.

## Supporting information


**Data S1:** acn370203‐sup‐0001‐TableS1‐S3‐FigureS1‐S10.docx.

## Data Availability

The data that support the findings of this study are available from the corresponding author upon reasonable request.
