# Supplementary material for: Systemic T Cell Receptor Profiling Reveals Adaptive Immune Activation and Potential Immune Signatures of Diagnosis and Brain Atrophy in Epilepsy
Source: Ann Clin Transl Neurol. 2025 Oct 7;13(2):296–309. doi: 10.1002/acn3.70203 (PMC12883695; doi:10.1002/acn3.70203)
Supplement: Supplementary file 1 — Data S1: acn370203‐sup‐0001‐TableS1‐S3‐FigureS1‐S10.docx. [file ACN3-13-296-s001.docx]

**Supplementary Tables**

**Supplementary Table S1. Demographics and clinical characteristics for study population**

|  | Total |  |  |  | Epilepsy subgroup | |  |  |
| --- | --- | --- | --- | --- | --- | --- | --- | --- |
|  | **Control**  **(*n* = 55)** | **Epilepsy**  **(*n* = 45)** | ***P* value** |  | **WCE**  **(*n* = 14)** | **DRE**  **(*n* = 22)** | **NIE**  **(*n* = 9)** | ***p* value** |
| Age, year | 44 (31, 57) | 39 (28, 49) | **0.019** |  | 34 (25, 53) | 38 (32, 48) | 50 (30, 52) | 0.7 |
| Sex |  |  | 0.4 |  |  |  |  | 0.6 |
| F | 30 (55%) | 21 (47%) |  |  | 6 (43%) | 12 (55%) | 3 (33%) |  |
| M | 25 (45%) | 24 (53%) |  |  | 8 (57%) | 10 (45%) | 6 (67%) |  |
| Age at onset, year |  | 20 (10, 38) |  |  | 19 (12, 49) | 15 (9, 31) | 45 (30, 52) | **0.008** |
| Duration of epilepsy, months |  | 60 (12, 276) |  |  | 42 (11, 180) | 252 (60, 420) | 6 (2, 31) | **<0.001** |
| Epilepsy type |  |  |  |  |  |  |  | >0.9 |
| TLE |  | 11 (77%) |  |  | 11 (79%) | 17 (77%) | 7 (78%) |  |
| Non-TLE |  | 10 (22%) |  |  | 3 (21%) | 5 (23%) | 2 (22%) |  |
| Epilepsy laterality |  |  |  |  |  |  |  | **0.026** |
| Unilateral |  | 25 (56%) |  |  | 11 (79%) | 12 (55%) | 2 (22%) |  |
| Bilateral/multifocal |  | 20 (44%) |  |  | 3 (21%) | 10 (45%) | 7 (78%) |  |
| Etiology |  |  |  |  |  |  |  | **<0.001** |
| Structural |  | 6 (13%) |  |  | 3 (21%) | 3 (14%) | 0 (0%) |  |
| Hippocampal sclerosis |  | 7 (16%) |  |  | 2 (14%) | 5 (23%) | 0 (0%) |  |
| FCD Ia |  | 1 (2%) |  |  | 0 (0%) | 1 (5%) | 0 (0%) |  |
| FCD IIa |  | 1 (2%) |  |  | 0 (0%) | 1 (5%) | 0 (0%) |  |
| DNET |  | 1 (2%) |  |  | 0 (0%) | 1 (5%) | 0 (0%) |  |
| Immune/  Post-infectious |  | 9 (20%) |  |  | 0 (0%) | 0 (0%) | 9 (100%) |  |
| Nonlesional/Unclear |  | 19 (42%) |  |  | 9 (64%) | 10 (45%) | 0 (0%) |  |
| Generalized epilepsy |  | 1 (2%) |  |  | 0 (0%) | 1 (5%) | 0 (0%) |  |
| Seizure frequency |  |  |  |  |  |  |  |  |
| Total frequency, per month |  | 4 (1, 8) |  |  | 1 (1, 6) | 5 (3, 15) | 2 (1, 5) | **0.032** |
| TCS frequency, per month |  | 0 (0, 0.5) |  |  | 0 (0, 0.5) | 0 (0, 1) | 0 (0, 0.5) | 0.93 |
| Non-TCS frequency, per month |  | 2 (0, 8) |  |  | 1 (0, 6) | 3.5 (1, 15) | 0 (0, 2) | 0.12 |
| No. ASM at baseline |  |  |  |  |  |  |  | **<0.001** |
| 0 |  |  |  |  | 8 (57%) | 0 (0%) | 1 (11%) |  |
| 1 |  |  |  |  | 4 (29%) | 1 (4.5%) | 2 (22%) |  |
| 2 |  |  |  |  | 1 (7.1%) | 4 (18%) | 3 (33%) |  |
| 3 |  |  |  |  | 0 (0%) | 10 (45%) | 2 (22%) |  |
| ≥4 |  |  |  |  | 1 (7.1%) | 7 (32%) | 1 (11%) |  |

Data are presented as *n* (%) or median (Q1, Q3), where Q1 and Q3 represent the first and third quantiles.

WCE = well-controlled epilepsy; DRE = drug-resistant epilepsy; NIE = neuroinflammation-associated epilepsy; TLE = temporal lobe epilepsy; FCD = focal cortical dysplasia; DNET = Dysembryoplastic neuroepithelial tumor; TCS = tonic-clonic seizure; ASM = antiseizure medication.

**Supplementary Table S2. TRAV, TRBV, and TRAJ genes showing significant differences in usage frequency between the groups**

| Gene | Category | Frequency in epilepsy, median (IQR) ^a^ | Frequency in control, median (IQR) ^a^ | Dominant group^b^ | *p* value | FDR |
| --- | --- | --- | --- | --- | --- | --- |
| TRAV26-2 | TRAV gene | 0.018 (0.009, 0.027) | 0.011 (0.005, 0.015) | Epilepsy | 3.79e-04 | 0.005 |
| TRAV4 | TRAV gene | 0.044 (0.035, 0.056) | 0.036 (0.028, 0.045) | Epilepsy | 0.003 | 0.015 |
| TRAV34 | TRAV gene | 0.003 (0.002, 0.004) | 0.003 (0.002, 0.003) | Epilepsy | 0.003 | 0.017 |
| TRAV10 | TRAV gene | 0.021 (0.018, 0.024) | 0.018 (0.016, 0.021) | Epilepsy | 0.006 | 0.024 |
| TRAV9-2 | TRAV gene | 0.057 (0.039, 0.069) | 0.046 (0.04, 0.052) | Epilepsy | 0.009 | 0.030 |
| TRAV14DV4 | TRAV gene | 0.017 (0.01, 0.02) | 0.021 (0.019, 0.026) | Control | 4.9e-05 | 0.001 |
| TRAV8-1 | TRAV gene | 0.009 (0.005, 0.014) | 0.014 (0.012, 0.017) | Control | 6.21e-05 | 0.001 |
| TRAV23DV6 | TRAV gene | 0.022 (0.014, 0.029) | 0.03 (0.025, 0.032) | Control | 4.67e-04 | 0.005 |
| TRAV29DV5 | TRAV gene | 0.051 (0.041, 0.058) | 0.059 (0.053, 0.062) | Control | 0.001 | 0.007 |
| TRAV3 | TRAV gene | 0.015 (0.009, 0.026) | 0.02 (0.017, 0.029) | Control | 0.001 | 0.007 |
| TRAV8-3 | TRAV gene | 0.012 (0.008, 0.025) | 0.021 (0.015, 0.027) | Control | 0.002 | 0.015 |
| TRAV17 | TRAV gene | 0.033 (0.03, 0.036) | 0.036 (0.033, 0.039) | Control | 0.006 | 0.024 |
| TRAV35 | TRAV gene | 0.017 (0.014, 0.019) | 0.019 (0.016, 0.021) | Control | 0.006 | 0.024 |
| TRAV41 | TRAV gene | 0.008 (0.006, 0.009) | 0.009 (0.008, 0.01) | Control | 0.011 | 0.035 |
| TRBV27 | TRBV gene | 0.039 (0.031, 0.045) | 0.032 (0.027, 0.036) | Epilepsy | 1.55e-04 | 0.007 |
| TRBV13 | TRBV gene | 0.005 (0.004, 0.006) | 0.004 (0.003, 0.005) | Epilepsy | 0.001 | 0.014 |
| TRBV9 | TRBV gene | 0.022 (0.02, 0.029) | 0.021 (0.018, 0.022) | Epilepsy | 0.006 | 0.032 |
| TRBV11-3 | TRBV gene | 0.005 (0.003, 0.007) | 0.004 (0.003, 0.005) | Epilepsy | 0.011 | 0.050 |
| TRBV19 | TRBV gene | 0.014 (0.009, 0.027) | 0.023 (0.02, 0.027) | Control | 0.001 | 0.013 |
| TRBV5-6 | TRBV gene | 0.016 (0.014, 0.021) | 0.019 (0.018, 0.021) | Control | 0.001 | 0.014 |
| TRBV5-5 | TRBV gene | 0.013 (0.01, 0.016) | 0.015 (0.014, 0.016) | Control | 0.002 | 0.017 |
| TRBV5-4 | TRBV gene | 0.008 (0.006, 0.014) | 0.011 (0.009, 0.017) | Control | 0.002 | 0.019 |
| TRBV14 | TRBV gene | 0.005 (0.004, 0.009) | 0.008 (0.007, 0.009) | Control | 0.003 | 0.020 |
| TRBV11-2 | TRBV gene | 0.018 (0.016, 0.02) | 0.021 (0.018, 0.024) | Control | 0.006 | 0.032 |
| TRBV12-3 | TRBV gene | 0.054 (0.045, 0.059) | 0.058 (0.053, 0.063) | Control | 0.010 | 0.048 |
| TRAJ34 | TRAJ gene | 0.027 (0.025, 0.029) | 0.025 (0.024, 0.026) | Epilepsy | 1.68e-04 | 0.007 |
| TRAJ32 | TRAJ gene | 0.018 (0.017, 0.018) | 0.017 (0.016, 0.018) | Epilepsy | 3.23e-04 | 0.007 |
| TRAJ39 | TRAJ gene | 0.029 (0.027, 0.031) | 0.026 (0.025, 0.028) | Epilepsy | 4.1e-04 | 0.007 |
| TRAJ27 | TRAJ gene | 0.018 (0.017, 0.019) | 0.018 (0.016, 0.019) | Epilepsy | 0.002 | 0.023 |

^a^Data are presented as median (Q1, Q3), where Q1 and Q3 represent the first and third quantiles.

^b^Based on whether the median frequency is higher in epilepsy

IQR = interquartile range; FDR = false discovery rate correction for multiple testing

**Supplementary Table S3. Spearman correlation analysis of seizure frequency and TCR diversity in patients excluding NIE and daily seizures**

|  | No adjustment | | | |  | Model 1^a^ |  |  | Model 2^b^ |  |  | Model 3^c^ |  |  | Model 4^d^ |  |  |  |
| --- | --- | --- | --- | --- | --- | --- | --- | --- | --- | --- | --- | --- | --- | --- | --- | --- | --- | --- |
|  | **Correlation coefficient** | | ***p* value^e^** | |  | **Correlation coefficient** | ***p* value^e^** |  | **Correlation coefficient** | ***p* value^e^** |  | **Correlation coefficient** | ***p* value^e^** |  | **Correlation coefficient** | ***p* value^e^** |  | **LASSO selected variables^f^** |
| Total seizure frequency (mo) | | |  | |  |  |  |  |  |  |  |  |  |  |  |  |  |  |
| TRA |  | |  | |  |  |  |  |  |  |  |  |  |  |  |  |  |  |
| Richness | **-0.413** | | **0.017*** | |  | **-0.413** | **0.019*** |  | **-0.459** | **0.008**** |  | **-0.415** | **0.020*** |  | **-0.464** | **0.007**** |  | PC1 |
| Shannon | -0.335 | | 0.057 | |  | -0.335 | 0.061 |  | **-0.416** | **0.018*** |  | -0.335 | 0.066 |  | **-0.378** | **0.036*** |  | PC1, U |
| D50 | **-0.407** | | **0.019*** | |  | **-0.406** | **0.021*** |  | **-0.464** | **0.007**** |  | **-0.407** | **0.023*** |  | **-0.471** | **0.007**** |  | PC1 |
| Clonality | 0.306 | | 0.084 | |  | 0.305 | 0.089 |  | **0.379** | **0.032*** |  | 0.306 | 0.094 |  | 0.334 | 0.071 |  | PC1, PC2, U |
| Prop_stim | **0.405** | | **0.019*** | |  | **0.405** | **0.022*** |  | **0.458** | **0.008**** |  | **0.405** | **0.024*** |  | **0.463** | **0.008**** |  | PC1 |
| TRB |  | |  | |  |  |  |  |  |  |  |  |  |  |  |  |  |  |
| Richness | **-0.458** | | **0.007**** | |  | **-0.460** | **0.008**** |  | **-0.505** | **0.003**** |  | **-0.464** | **0.009**** |  | **-0.476** | **0.008**** |  | PC1, U, S |
| Shannon | -0.341 | | 0.052 | |  | -0.347 | 0.052 |  | **-0.396** | **0.025*** |  | **-0.347** | **0.056** |  | **-0.358** | **0.048*** |  | PC1, U |
| D50 | **-0.455** | | **0.008**** | |  | **-0.458** | **0.008**** |  | **-0.503** | **0.003**** |  | **-0.460** | **0.009**** |  | **-0.510** | **0.003**** |  | PC1 |
| Clonality | 0.334 | | 0.057 | |  | 0.342 | 0.056 |  | **0.391** | **0.027*** |  | 0.342 | 0.060 |  | 0.351 | 0.053 |  | PC1, U |
| Prop_stim | **0.433** | | **0.012*** | |  | **0.437** | **0.012*** |  | **0.475** | **0.006**** |  | **0.439** | **0.014*** |  | **0.437** | **0.016*** |  | PC1, U, S |
| Non-TCS frequency (mo) | |  | |  |  |  |  |  |  |  |  |  |  |  |  |  |  |  |
| TRA |  | |  | |  |  |  |  |  |  |  |  |  |  |  |  |  |  |
| Richness | **-0.453** | | **0.008**** | |  | **-0.454** | **0.009**** |  | **-0.424** | **0.016*** |  | **-0.454** | **0.010*** |  | **-0.421** | **0.021*** |  | PC1, U, S |
| Shannon | **-0.397** | | **0.022*** | |  | **-0.403** | **0.022*** |  | **-0.357** | **0.045*** |  | **-0.403** | **0.025*** |  | -0.341 | 0.066 |  | PC1, PC2, U |
| D50 | **-0.465** | | **0.006**** | |  | **-0.469** | **0.007**** |  | **-0.435** | **0.013*** |  | **-0.469** | **0.008**** |  | **-0.436** | **0.013*** |  | PC1 |
| Clonality | **0.373** | | **0.033*** | |  | **0.380** | **0.032*** |  | 0.330 | 0.065 |  | **0.381** | **0.034*** |  | 0.316 | 0.095 |  | PC1, PC2, U, S |
| Prop_stim | **0.465** | | **0.006**** | |  | **0.467** | **0.007**** |  | **0.435** | **0.013*** |  | **0.467** | **0.008**** |  | **0.432** | **0.015*** |  | PC1, U |
| TRB |  | |  | |  |  |  |  |  |  |  |  |  |  |  |  |  |  |
| Richness | **-0.485** | | **0.004**** | |  | **-0.494** | **0.004**** |  | **-0.459** | **0.008**** |  | **-0.496** | **0.005**** |  | **-0.458** | **0.013*** |  | PC1. PC2, U, S |
| Shannon | **-0.388** | | **0.026*** | |  | **-0.405** | **0.021*** |  | **-0.351** | **0.049*** |  | **-0.405** | **0.024*** |  | -0.345 | 0.057 |  | PC1, U |
| D50 | **-0.479** | | **0.005**** | |  | **-0.489** | **0.005**** |  | **-0.452** | **0.009**** |  | **-0.489** | **0.005**** |  | **-0.452** | **0.009**** |  | PC1 |
| Clonality | **0.390** | | **0.025*** | |  | **0.413** | **0.019*** |  | **0.353** | **0.047*** |  | **0.412** | **0.021*** |  | 0.348 | 0.055 |  | PC1, U |
| Prop_stim | **0.471** | | **0.006**** | |  | **0.485** | **0.005**** |  | **0.445** | **0.011*** |  | **0.485** | **0.006**** |  | **0.445** | **0.011*** |  | PC1 |

^a^Model 1, partial correlation adjusted for age.

^b^Model 2, partial correlation adjusted for age at onset.

^c^Model 3, partial correlation adjusted for age and sex.

^d^Model 4, partial correlation for LASSO-selected variables. For age-related variables (age, age at onset, duration of epilepsy), PC1 and PC2 (accounting for 99% of the variance) were used after principal component analysis.

^e^ **p* < 0.05; ***p* < 0.01; ****p* < 0.001.

^f^Seizure frequency was always selected among LASSO selected variables and therefore omitted from the table.

TCR = T cell receptor; NIE = neuroinflammation-associated epilepsy; LASSO = least absolute shrinkage and selection operator; TRA = T cell receptor α chain; TRB = T cell receptor β chain; PC = principal component; U = unilateral epileptic foci (compared to bilateral/multifocal foci); S = sex; Shannon = Shannon diversity index; D50 = the minimum number of clones accounting for 50% of the repertoire; Prop_stim = proportion of highly stimulate clones; TCS = tonic-clonic seizure.

**Supplementary Figures**

**
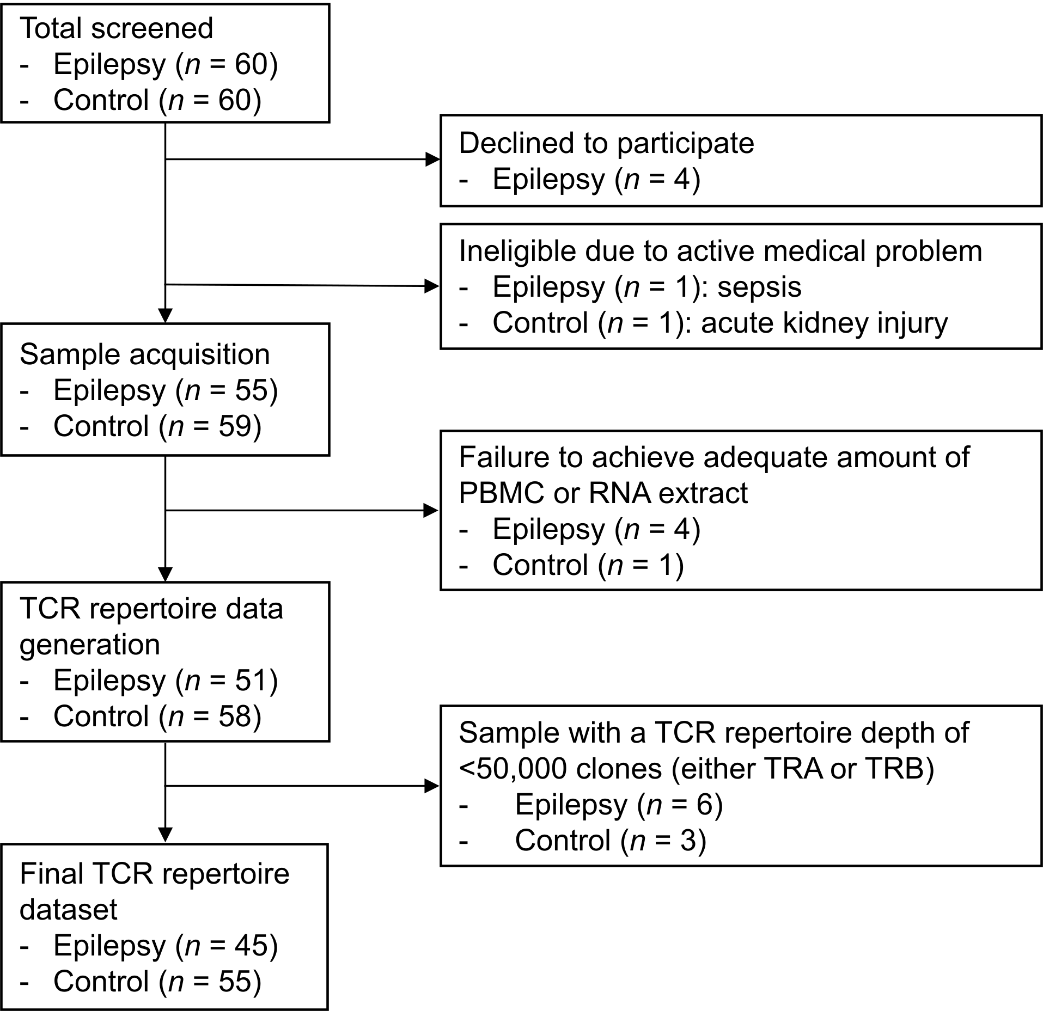
**

**Supplementary Figure S1. Flow Diagram for study inclusion and exclusions.** PBMC = peripheral blood mononuclear cell; TCR = T cell receptor; TRA = T cell receptor α chain; TRB = T cell receptor β chain.


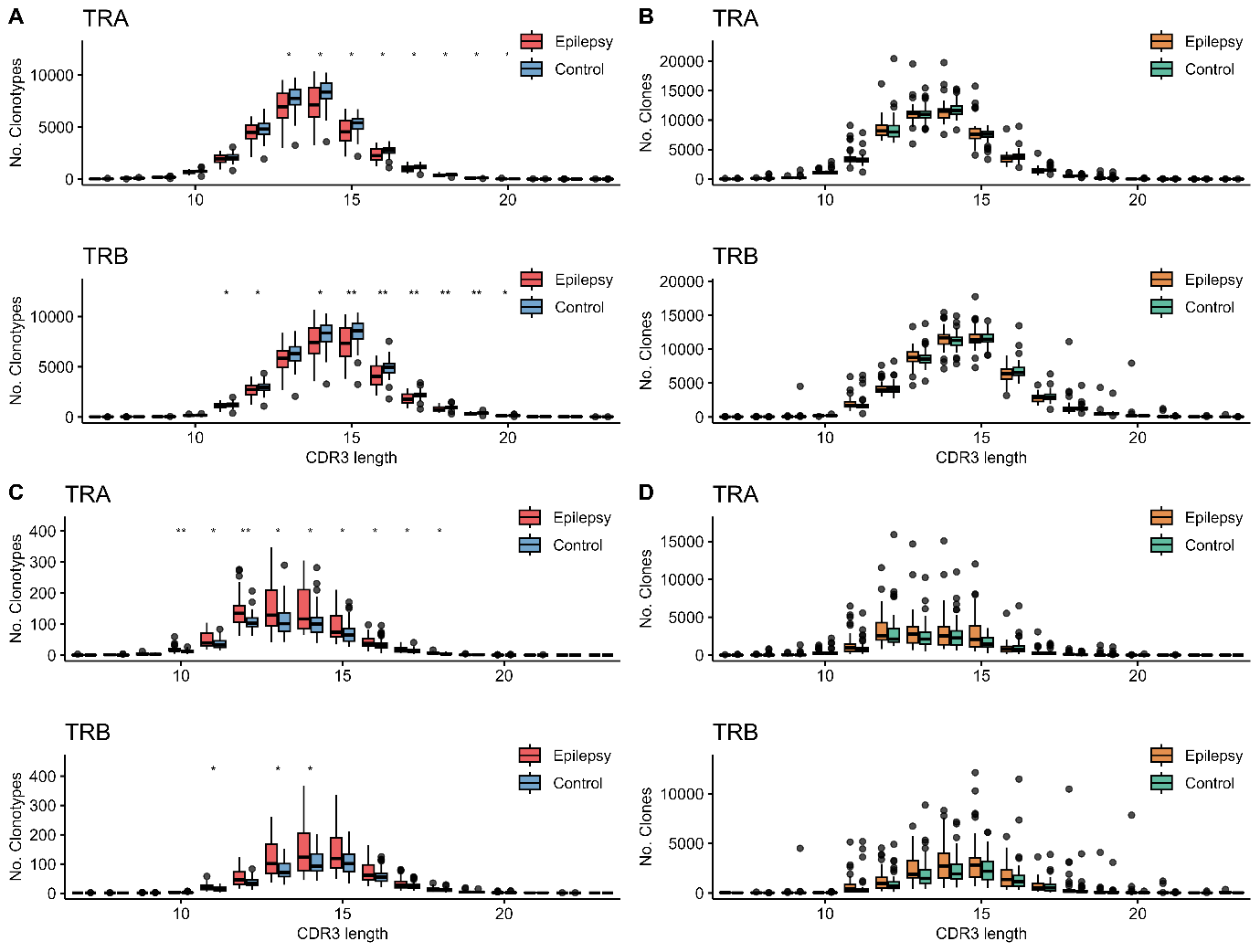


**Supplementary Figure S2. Clonal distributions of the TCR by CDR3 length.** (**A**) Comparison of the number of clonotypes for each CDR3 length between epilepsy and control groups, separately for TRA and TRB. (**B**) Comparison of the clone counts for each CDR3 length between epilepsy and control groups, separately for TRA and TRB. (**C, D**) Distribution of the number of clonotypes (**C**) and clones (**D**) for subsets of TCR clonotypes with clone counts greater than 4. Adjusted *p* values are shown for each CDR3 length; statistical significance is indicated as follows: **p* < 0.05, ***p* < 0.01. TCR = T cell receptor; CDR3 = complementarity-determining region 3; TRA = T cell receptor α chain; TRB = T cell receptor β chain.


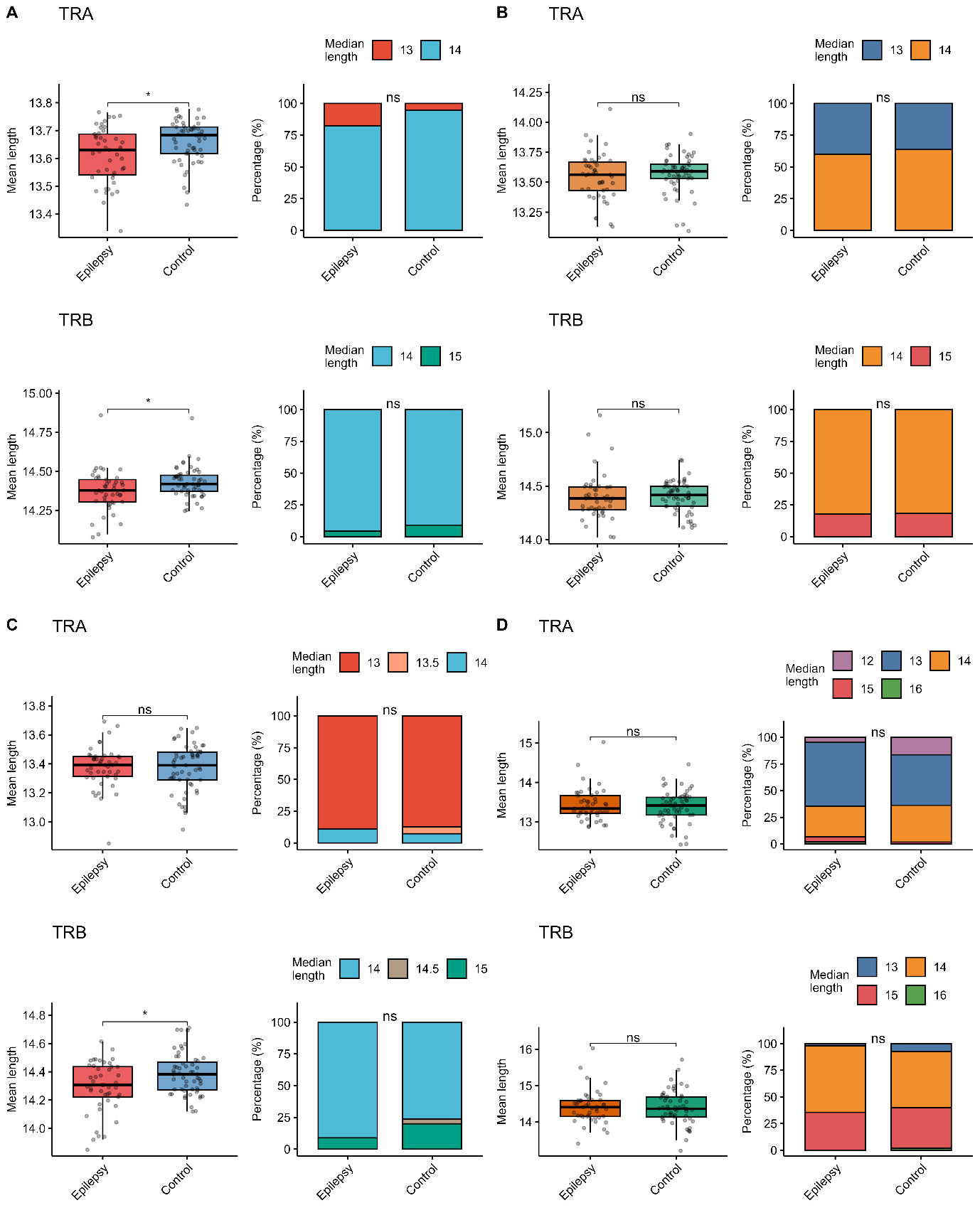
**Supplementary Figure S3. Comparison of mean and median CDR3 lengths in each TCR repertoire.** (**A, B**) Comparison of mean CDR3 lengths (boxplots) and median CDR3 length distributions (stacked bar plots) between epilepsy and control groups, analyzed separately for unique clonotypes (**A**) and for all clones (**B**) in each TCR repertoire (TRA and TRB). (**C, D**) Comparison of mean and median CDR3 lengths for clonotypes with clone counts greater than 4, shown separately for unique clonotypes (**C**) and for all clones (**D**). Statistical significance is indicated as follows: “ns” represents no significant difference, **p* < 0.05. CDR3 = complementarity-determining region 3; TCR = T cell receptor; TRA = T cell receptor α chain; TRB = T cell receptor β chain.

**
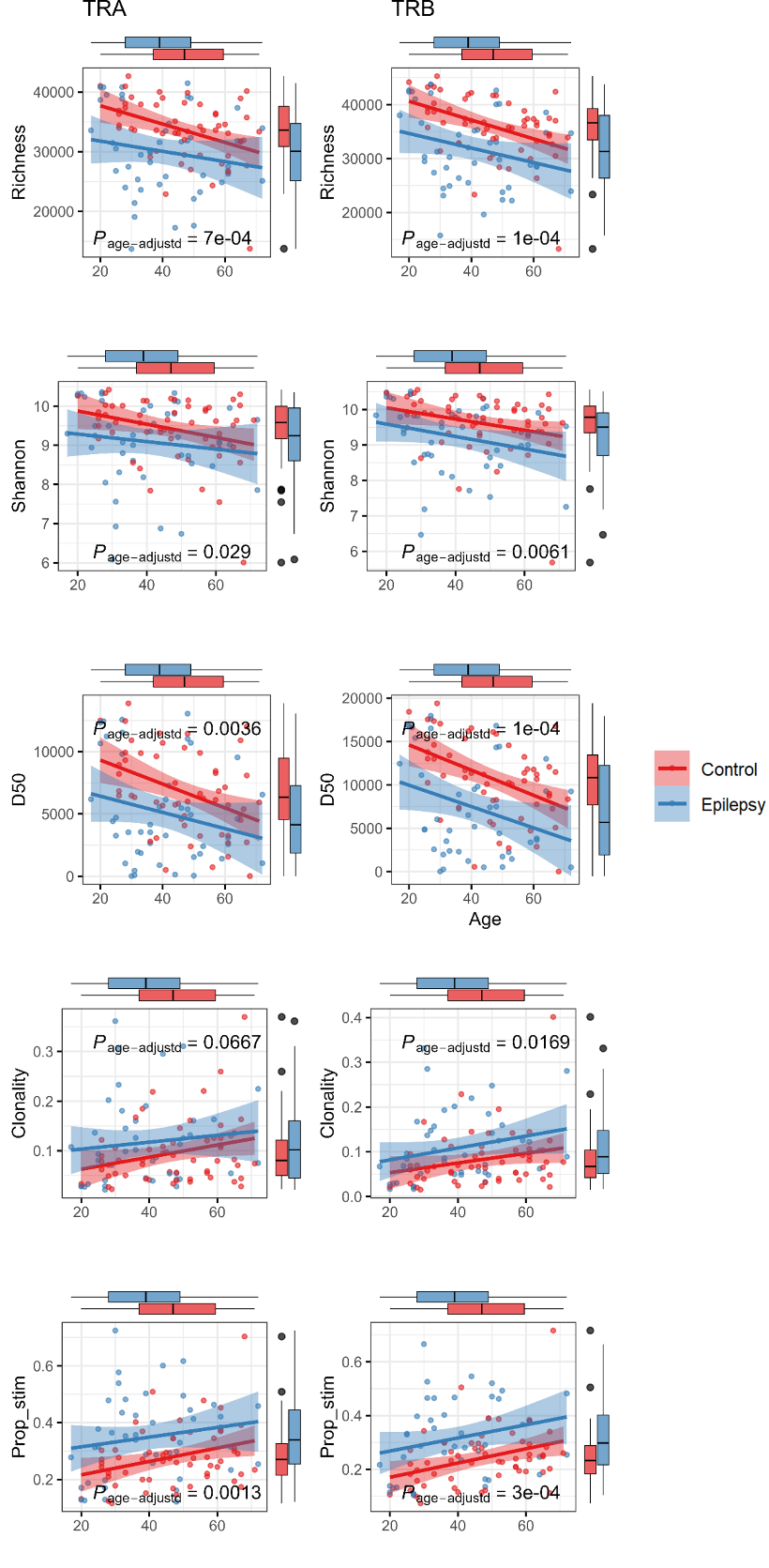
**

**Supplementary Figure S4. Epilepsy-associated reduction in TCR diversity independent of age.** Scatterplots illustrating the relationship between age and various TCR diversity metrics, with separate regression lines for the epilepsy and control groups. Each plot shows a noticeable gap between the two regression lines, indicating statistically significant differences between groups after accounting for age effects. The age-adjusted *P* value is displayed within each plot. TCR = T cell receptor; TRA = T cell receptor α chain; TRB = T cell receptor β chain; Shannon = Shannon diversity index; D50 = the minimum number of clones accounting for 50% of the repertoire; Prop_stim = proportion of highly stimulated clones.

**
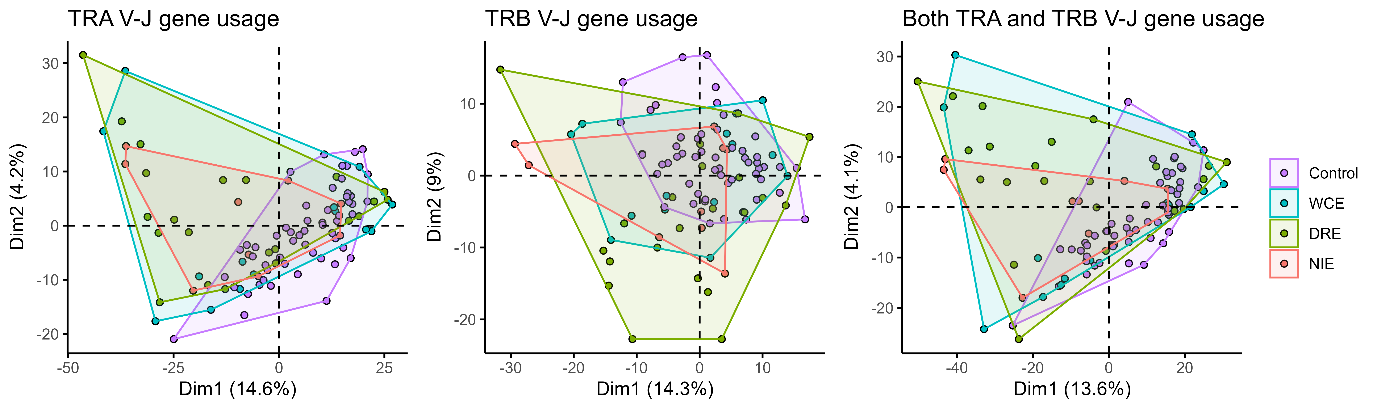
**

**Supplementary Figure S5. Principal component analysis of V-J gene usage frequencies in epilepsy subgroups and controls.** Principal component analysis plot of the first and second principal components based on combined V-J gene usage frequencies, with points grouped into control, WCE, DRE, and NIE. Axis labels indicate the principal components and the percentage of variance explained by each. TRA = T cell receptor α chain; TRB = T cell receptor β chain; WCE = well-controlled epilepsy, DRE = drug-resistant epilepsy; NIE = new-onset immune epilepsy.


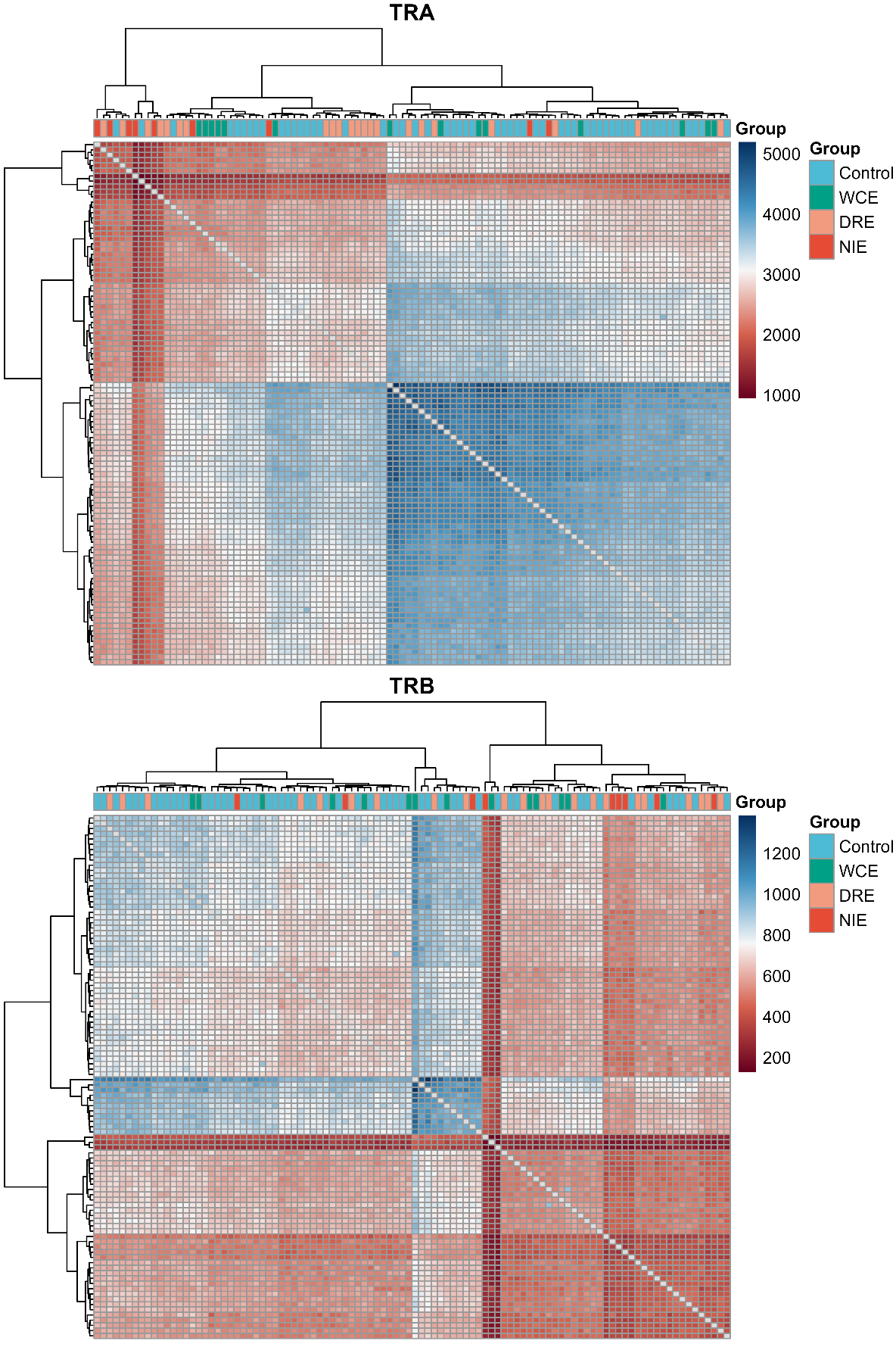
**Supplementary Figure S6. Heatmaps showing the number of shared TCR clonotypes between individual samples across epilepsy subgroups and controls.** Hierarchical clustering was applied to both rows and columns of each heatmap for the TRA and TRB repertoires. Samples are annotated by four subgroups. TRA = T cell receptor α chain; TRB = T cell receptor β chain; WCE = well-controlled epilepsy, DRE = drug-resistant epilepsy; NIE = new-onset immune epilepsy.


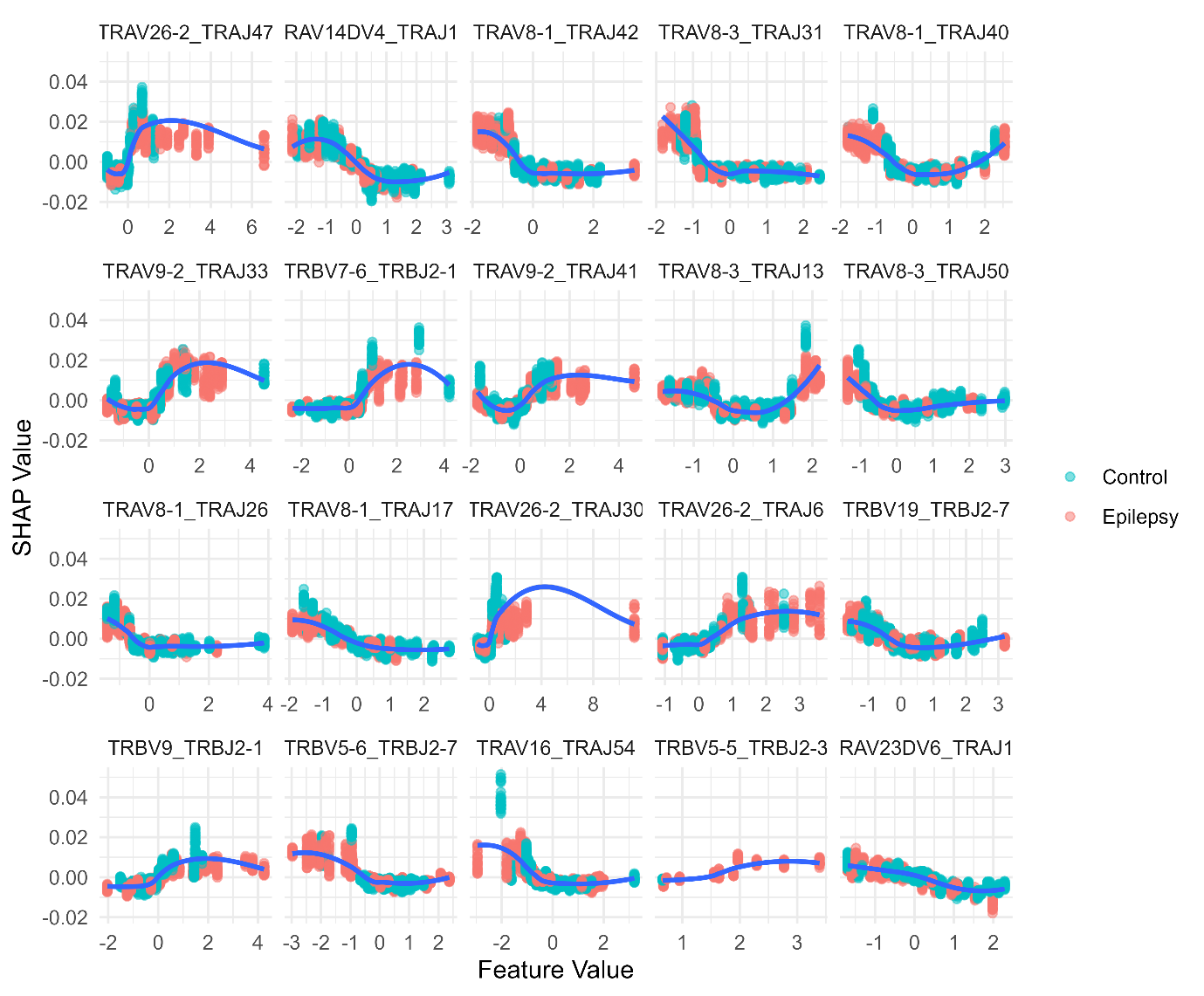


**Supplementary Figure S7. SHAP dependence plot for top 20 features.** Scatterplots showing the relationship between standardized feature values and SHAP values for the top 20 most important features. Each point represents a sample, with color indicating the group (epilepsy or control). A local regression (loess) line is fitted to visualize the dependence between the feature value and SHAP value. The plots are faceted by feature name. SHAP = Shapley Additive Explanations.


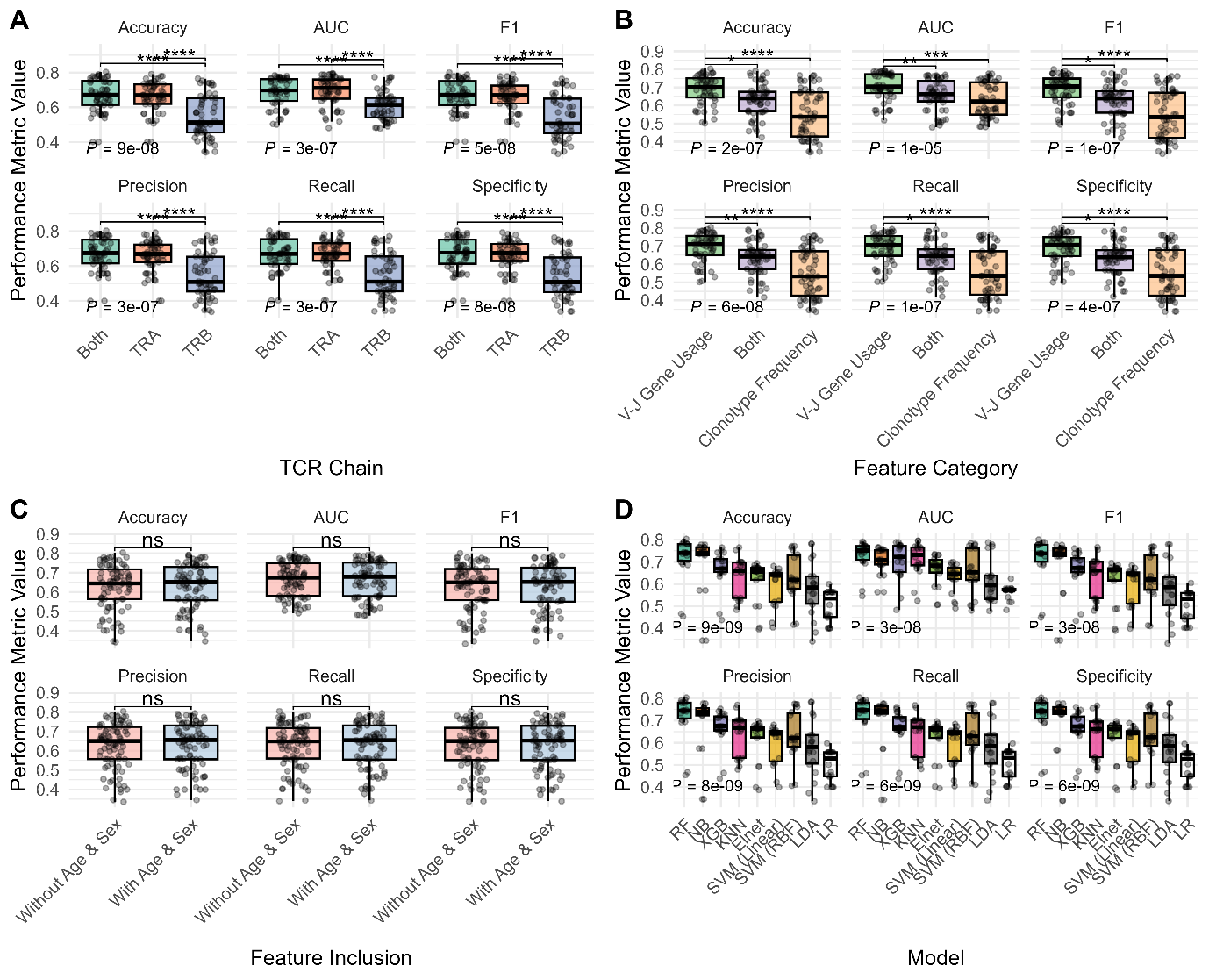


**Supplementary Figure S8. Comparison of performance based on included features and machine learning models.** (**A**) Performance differences depending on the inclusion of TRA, TRB or both chain-related features. (**B**) Performance differences depending on the inclusion of clonotype frequency, combined V-J gene usage or both. (**C**) Performance differences depending on the inclusion of age and sex information as features for machine learning. (**D**) Performance differences across different machine learning models. Statistical significance is indicated as follows: "ns" represents no significant difference, **p* < 0.05, ***p* < 0.01, ****p* < 0.001, *****p* < 0.0001. Significance levels are Bonferroni-adjusted based on post-hoc tests for all possible pairwise comparisons. Pairwise comparisons that yielded no significant differences are not displayed in panels (**A**) and (**B**). Global Kruskal-Wallis test *p* values are also presented for (**A**), (**C**) and (**D**). AUC = area under the receiver operating characteristic curve; F1 = F1 score; RF = random forest model; NB = naïve Bayes model; XGB = XGBoost model; KNN = k-nearest neighbors model; Elnet = elastic net model; SVM (Linear) = support vector machine with a linear kernel; SVM (RBF) = support vector machine with a radial basis function kernel; LDA = linear discriminant analysis model; LR = logistic regression model; TCR = T cell receptor; TRA = T cell receptor α chain; TRB = T cell receptor β chain.


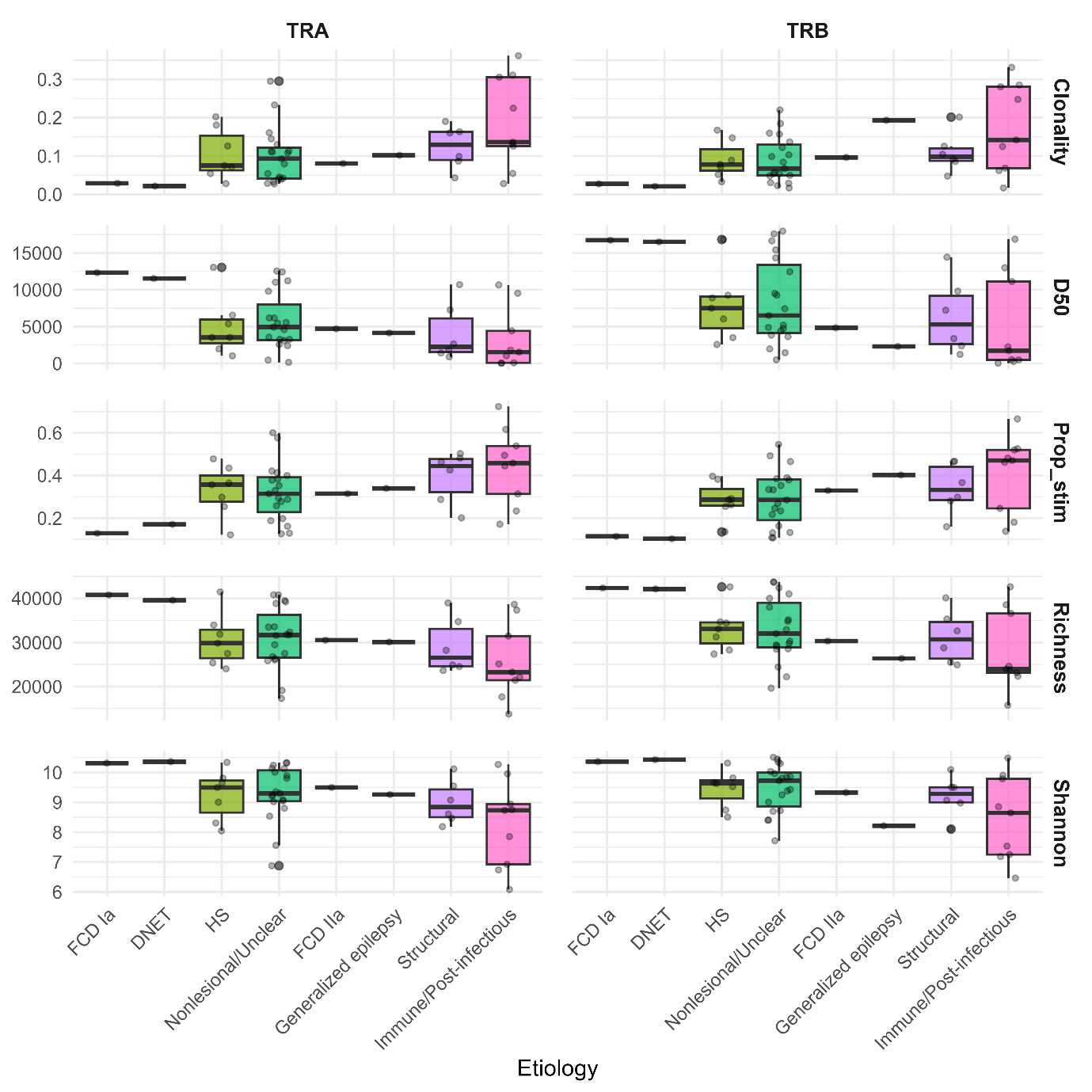


**Supplementary Figure S9. TCR repertoire diversity across epilepsy etiologies.** TRA = T cell receptor α chain; TRB = T cell receptor β chain; Shannon = Shannon diversity index; D50 = the minimum number of clones accounting for 50% of the repertoire; Prop_stim = proportion of highly stimulated clones; FCD = focal cortical dysplasia; DNET = Dysembryoplastic neuroepithelial tumor; HS = hippocampal sclerosis.


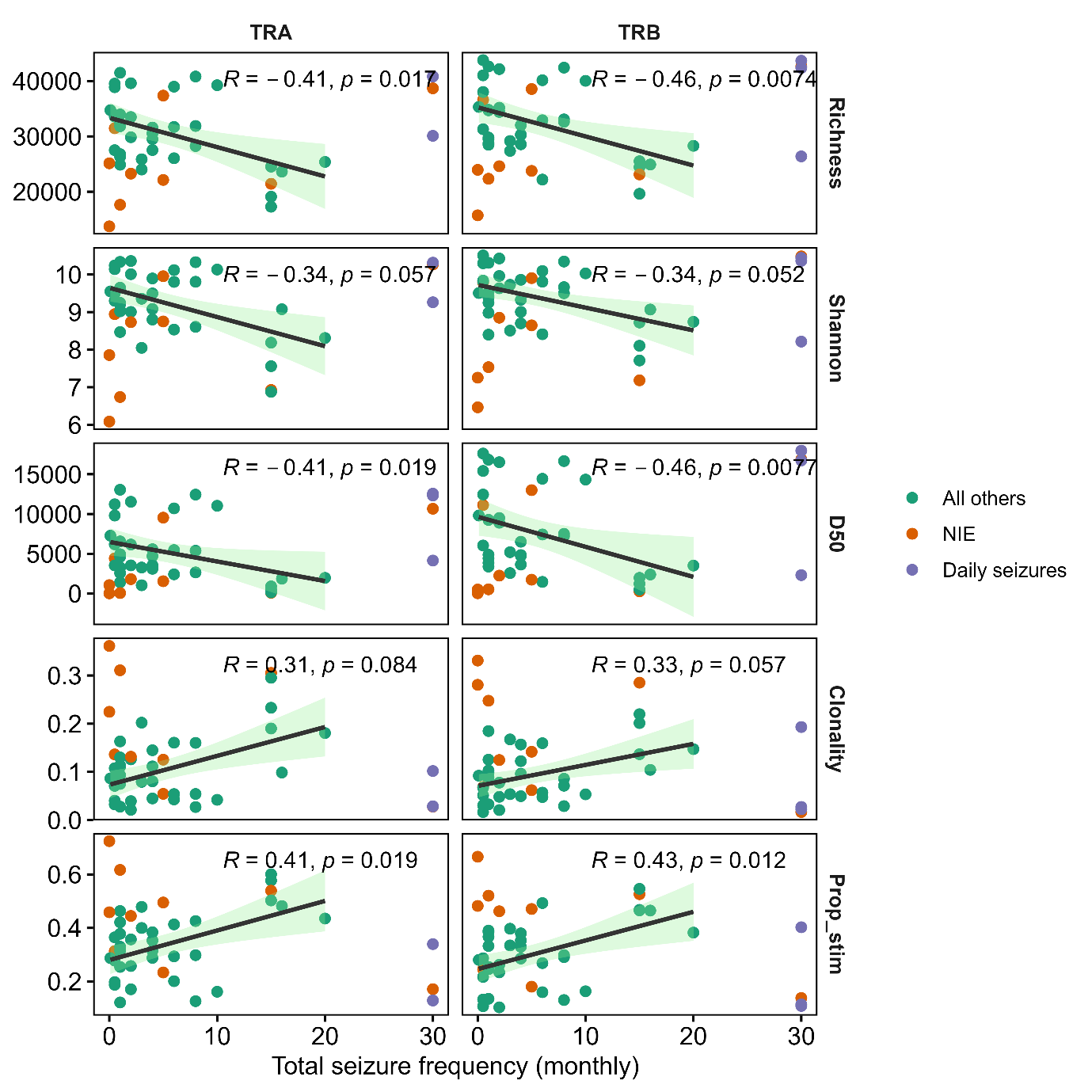


**Supplementary Figure S10. Scatterplots for seizure frequency and TCR repertoire diversity metrics.** The scatterplots show the relationship between seizure frequency and TCR repertoire diversity metrics across three groups. Outliers that show a marked decrease in TCR repertoire diversity with clonal proliferation in low seizure frequency mainly consist of patients with NIE. Regression line and confidence intervals specifically for subjects not categorized in NIE and without daily seizures (all others) are displayed along with Spearman correlation coefficients with *p* values (unadjusted). TCR = T cell receptor; NIE = neuroinflammation-associated epilepsy; TRA = T cell receptor alpha chain; TRB = T cell receptor beta chain; Shannon = Shannon diversity index; D50 = the minimum number of clones accounting for 50% of the repertoire; Prop_stim = proportion of highly stimulated clones.
